# Environment and culture shape both the colour lexicon and the genetics of colour perception

**DOI:** 10.1038/s41598-021-98550-3

**Published:** 2021-09-27

**Authors:** Mathilde Josserand, Emma Meeussen, Asifa Majid, Dan Dediu

**Affiliations:** 1 Laboratoire Dynamique Du Langage UMR 5596, Université Lumière Lyon 2, 14 Avenue Berthelot, 69363 Lyon Cedex 07, France; 2 Nijmegen, The Netherlands; 3 University of York, Heslington, York, YO10 5DD UK

**Keywords:** Human behaviour, Colour vision, Cultural evolution

## Abstract

Many languages express ‘blue’ and ‘green’ under an umbrella term ‘grue’. To explain this variation, it has been suggested that changes in eye physiology, due to UV-light incidence, can lead to abnormalities in blue-green color perception which causes the color lexicon to adapt. Here, we apply advanced statistics on a set of 142 populations to model how different factors shape the presence of a specific term for blue. In addition, we examined if the ontogenetic effect of UV-light on color perception generates a negative selection pressure against inherited abnormal red-green perception. We found the presence of a specific term for blue was influenced by UV incidence as well as several additional factors, including cultural complexity. Moreover, there was evidence that UV incidence was negatively related to abnormal red-green color perception. These results demonstrate that variation in languages can only be understood in the context of their cultural, biological, and physical environments.

## Introduction

One of the major puzzles facing the language sciences is how to best account for the factors and processes shaping the observed patterns of linguistic diversity today. The color lexicon is particularly interesting from this perspective. The long-standing emphasis has been on establishing universals^1^. Recent work shows that universal tendencies in color naming systems arise from perceptual structure^2^ and communicative needs^3–5^. At the same time, it is just as important to be able to characterize and explain variation found in color terminologies^6^. Languages differ in the number of basic color terms and which distinctions exactly are recognized for naming. Some languages only lexicalize ‘black’, ‘white’, and ‘red’; while others make many more distinctions, including ‘green’ and ‘blue’.

Blue is a particularly interesting color term to study since there are competing explanations for why it would emerge as a word. According to one class of theories, color terms emerge from salient features of the environment: for blue this would include water bodies, such as the sea, lakes, or rivers, as well as visibility of a blue sky which is affected by climate and humidity. That is, the ecological environment (such as the presence of lakes) may be a factor relevant in shaping the color lexicon^7–10^, suggesting that languages spoken in different environments may categorize the color continuum in different ways. An alternative account appeals to cultural practices, such as the presence of dyeing technologies^1,11,12^ and industrialization^3^. On this account, the emergence of blue comes about in those societies where blue dyes are used to color artifacts. More generally, it has been predicted that complex color terminologies (including the presence of blue) emerge in more complex societies^12–14^.

**Figure 1:**
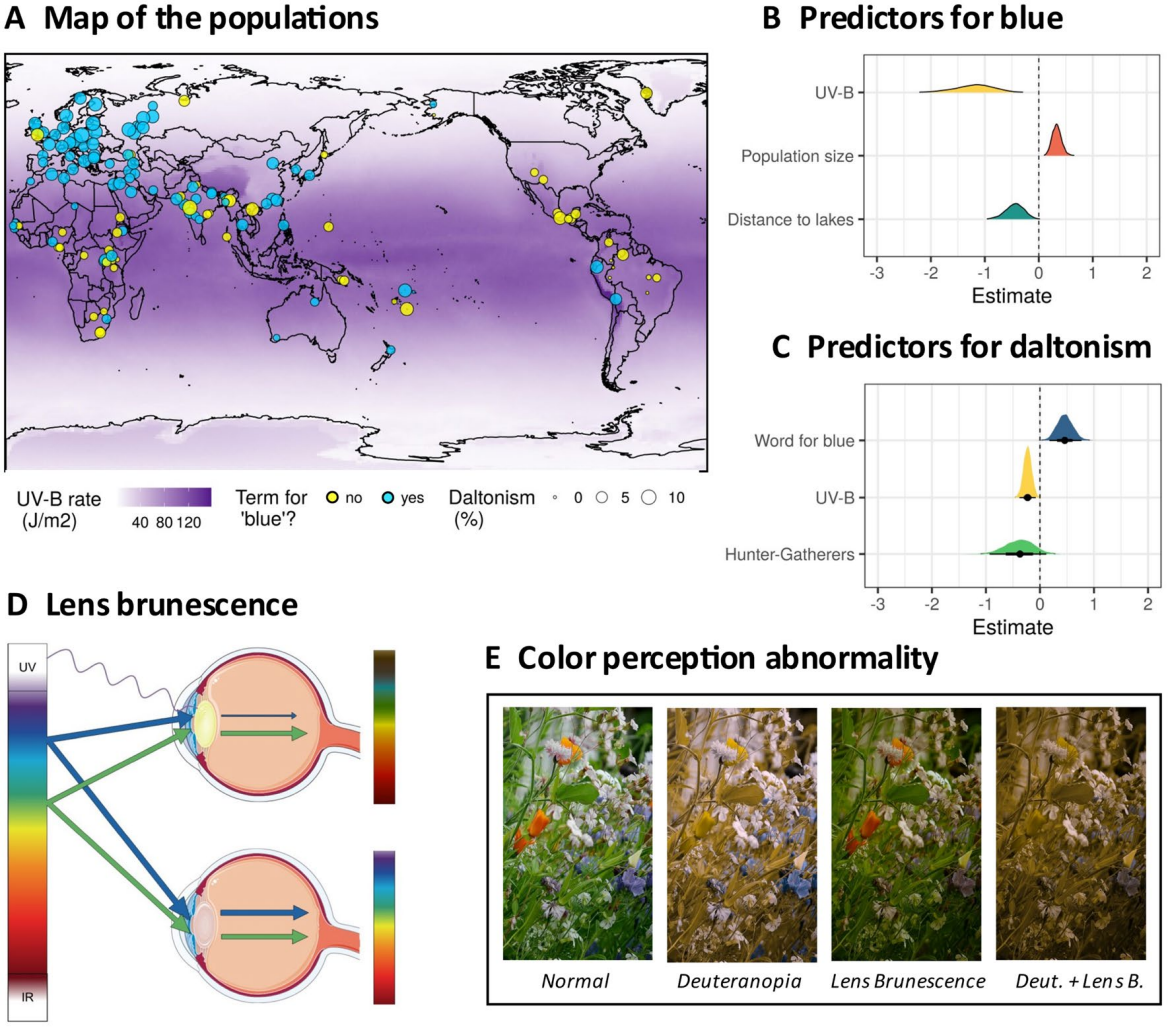
(Panel **A**): Map of languages and populations in our data. Each circle represents one language and corresponding population, with the presence (blue) or absence (yellow) for a specific term for ’blue’. The circle radius is proportional to the population frequency of red/green abnormal color perception. The intensity of the background magenta is proportional to the incidence of UV-B radiation as given by the NASA *Total Ozone Mapping Spectrometer* (TOMS) for the year 1998. This map was generated with the package maps in R version 4.0.5^59^, which uses public domain data from the *Natural Earth* project https://www.naturalearthdata.com. (Panel **B**): Posterior density plots of the best predictors of the presence of a word for blue across Bayesian regression models. (Panel **C**): Posterior density plots of the best predictors of the frequency of red-green abnormal color perception across Bayesian regression models. The plots in panels (**B**) and (**C**) are based on the analyses detailed in the “Results” section and were generated using R^59^. (Panel **D**): The lens brunescence hypothesis, as detailed in this paper. UV rays cause the lens to opacify and become yellower (top), filtering blue light, so that the perceived spectrum (on the right) contains less blue and more green, compared to the non-affected eye (bottom). (Panel **E**): A simulation of different types of color perception abnormalities. From left to right: normal vision; one type of red-green abnormal color perception (deuteranopia); strong lens brunescence (yellow filter and darkening of the image); both lens brunescence and deuteranopia, hypothesized to result in decreased biological fitness relative to other color vision types. Background image cropped from a photo by Joydeep Pal on *Unspalsh* (https://unsplash.com/photos/i6kccimZz_8) under a free-to-use license (https://unsplash.com/license). For deuteranopia we used *Coblis—The Color BLIndness Simulator* (https://www.color-blindness.com/coblis-color-blindness-simulator/) and checked the results with the *Colorblind Web Page Filter* (https://www.toptal.com/designers/colorfilter). The lens brunescence filter is inspired by^30^: we used Adobe Lightroom classic version 10.0 with a yellow filter and darkening to simulate a Kodak yellow filter. For more details, see the “Results”, “Materials and Methods” and Supplementary Materials.

A third explanation for the emergence of blue is grounded in physiology^15,16^. It has been suggested that increased exposure to ultraviolet light, specifically to UV-B, affects the physiology of the lens of the eye^17–22^, increasing, in the long term, lens opacity^23,24^ and yellow pigmentation density inside the lens^25^, which affects in turn how light reaches the retina (Fig. 1 Panel D). This process of lens brunescence, where yellow pigments absorb short-wavelength (blue) before reaching the retina, may reduce the ability to perceive the blue part of the color spectrum^26–29^ (Fig. 1 Panel E), increasing the probability that a single ‘grue’ term combining ’blue’ and ’green’ is used instead. That is, there is no distinct lexicalization of ‘blue’^16,30^. Lens brunescence is driven by environmental exposure to UV-B radiation which is related to *latitude* (locations closer to the equator receive more sunlight than those closer to the poles), *altitude* (higher elevations have less ozone filtering), *climate* and *humidity* (cloud coverage), *vegetation* (close versus open environments), and *culture* (amount and type of outdoor activity). This predicts that in populations with high exposure to UV-B radiation, the persistent loss of sensitivity to blue in adults across generations should generate negative pressure against the development of a ‘blue’ category or a tendency to lose it, while in populations with low exposure to UV-B incidence, other processes may proceed unhindered enabling the emergence and retention of ‘blue’. If true, we should observe a statistical tendency for fewer languages with a dedicated word for blue at high UV-B incidence (particularly close to the equator).

Previous cross-cultural analyses linking UV-B incidence and the presence of ‘blue’^16^ may have been biased by the lack of control for various confounds (especially language contact and inheritance from a shared proto-language^31^), exclusion of possible alternative explanatory factors (such as cultural complexity, environmental influences), lack of modeling of causal pathways, and the statistical methods used. Therefore, we combine multiple explanatory factors and potential confounds, including language contact generally (since specific data about the borrowing of words for blue is not available for most languages under consideration) and language family, subsistence strategy and population size (as proxies for cultural complexity^32,33^ as systematic, quantitative data about cultural artefacts and dyeing techniques across cultures does not exist), latitude (which partly determines UV-B incidence), climate, ecology and distance to large bodies of water (as proxies for various environmental influences) using a comprehensive database and advanced statistical methods. In doing so, we not only test the role of UV-B light on the development of the color lexicon, but also explicate the extent to which additional hypothesized factors shape the presence of a specific term for blue, and where relevant through which causal pathways specifically.

Environmentally-induced (or acquired) abnormal color perception is not the only type of color deficiency. Notably, inherited red-green color-blindness has a relatively simple genetic basis where certain alleles at the opsin genes on the X chromosome encode for abnormal pigments, resulting in a higher incidence of abnormal color perception in males specifically affecting the low (red) and mid (green) frequencies of the visual spectrum (Fig. 1 Panel E). It has previously been suggested that a subsistence strategy based on hunting and gathering might generate selective pressures against red-green abnormal color perception due to the high survival relevance of these colors in the wild, while the transition to agriculture and, in particular the industrial revolution, may have greatly relaxed these pressures^34,35^.

Moreover, these two broad types of abnormal color perception could interact with each other. At the individual level, for somebody already affected by a relatively profound red-green deficiency, also acquiring lens brunescence would have devastating effects on the ability to use color (Fig. 1 Panel E), and may affect survival and possibly the ability to produce and raise offspring. This predicts that, despite their different and a priori independent causal mechanisms (environmental exposure vs genetic), a negative correlation between the two could emerge across evolutionary time, in that there may be stronger selective pressure against the alleles responsible for red-green abnormal color perception in populations with high UV-B exposure, leading across generations to an overall lower frequency of such inherited abnormalities in these populations^9^. Here, we not only test the hypothesis that there is a negative relationship between UV-B incidence and the population frequency of red-green color deficiency using advanced methods and controls, we also include the potential influence of subsistence strategy.

In summary, we test two main hypotheses, one linking the existence of a dedicated word for blue to UV-B incidence while simultaneously controlling for other factors, and a second concerning the selective pressures against abnormal red-green vision in different environments. We do so on a large dataset of 142 populations (Fig. 1 Panel A) speaking languages from 32 families across the world, for which we collected information about the existence of a dedicated word for blue, the incidence of ultraviolet light, and the frequency of red-green color deficiency, as well as data on elevation, population size, subsistence strategy, climate, ecology, distance to large bodies of water, and several additional variables concerning the populations and physical environments they inhabit (see Methods and Supplementary Materials). We used Bayesian hierarchical regression, mediation and path analysis, and various machine learning techniques, informed by previous research and causal modeling, to specifically test these hypotheses in a comprehensive manner. We found that both hypotheses were by and large supported, but importantly they only captured part of the overall picture (Fig. 1 Panels B and C). While variation in UV-B incidence (ultimately due to variation in latitude) was the most important predictor of ‘blue’, population size and distance to large bodies of standing water also played a role, with their effects mediated by climate and subsistence strategy. Likewise, there was a lower frequency of people with red-green color deficiency in populations closer to the equator (i.e., with more lens brunescence), and whose languages lacked a dedicated word for blue.

## Results

### UV-B incidence predicts the existence of a specific word for blue

Here we tested the hypothesis that the incidence of UV (in particular, UV-B) predicts the existence of a specific word for blue, taking into consideration various potential confounds and causal pathways.

Both UV-A and UV-B affected *blue* negatively (here, *blue* is the dichotomous variable ‘is there a specific term for blue in the language?’, italics indicate variable names; for more information about the variables, see Methods): UV-A: $\beta = -1.03$, 95%HDI = $[-1.65, -0.38]$, $p(\beta =0) = 0.0154$, $p(\beta <0) = 1$; UV-B:$\beta = -1.12 [-1.75, -0.55]$, $p(\beta =0) = 0.0128$, $p(\beta <0) = 1$. Please note that for such Bayesian regression results we report the slope, $\beta$, of the fixed effect(s) of interest in terms of their mean and 95% HDI (Highest Density Interval), as well as specific tests that capture the posterior probability that the hypothesis is true (i.e., they should be interpreted directly, and not as frequentist *p*-values in terms of the probability of seeing such a result were the null hypothesis true); also, while directional hypotheses should be *a priori* motivated, point hypothesis do not have the same requirement. However, as UV-A and UV-B were highly multicollinear ($VIF_{mean UVA} = 36.3$, $VIF_{mean UVB} = 36.3$) and UV-A fitted the data worse than UV-B ($BF = 0.31$, $LOO = -1.29\pm 0.77$, $WAIC = -1.13\pm 0.77$, $KFOLD = -1.20\pm 1.63$; for such comparisons between two models, $m_{1}$ and $m_{2}$, we report the Bayes Factor, BF, ideally < 0.033 or > 10, as well as the LOO, WAIC and KFOLD as the difference in the Expected Log pointwise Predictive Density, ELPD, between the two models and its standard error, for which the absolute difference should be larger than the standard error; ideally, all these criteria should agree, but they capture *a priori* different aspects of model comparison^36^), we retained only UV-B in the following analyses. The variable *blue* was positively affected by *latitude* ($\beta = 4.39 [0.82, 8.01]$, $p(\beta =0) = 0.068$, $p(\beta <0) = 0.99$), *population size* ($\beta = 0.31 [0.16, 0.48]$, $p(\beta =0) = 3.8 \times 10^{-7})$ and *climate PC1* ($\beta = 0.99 [0.40, 1.57]$, $p(\beta =0) = 0.035$), and negatively by *distance to lakes* ($\beta = -0.56 [-0.87, -0.27]$, $p(\beta =0) = 0.0074$). Furthermore, 4 of the 10 dimensions, resulting from the multidimensional scaling (MDS) of the matrix of genetic distances ($F_{ST}$^37^) between all pairs of populations, also affected *blue* (positively for *genetic D7*, and negatively for *genetic D1*, *genetic D4* and *genetic D6*; see Supplementary Materials).

**Figure 2:**
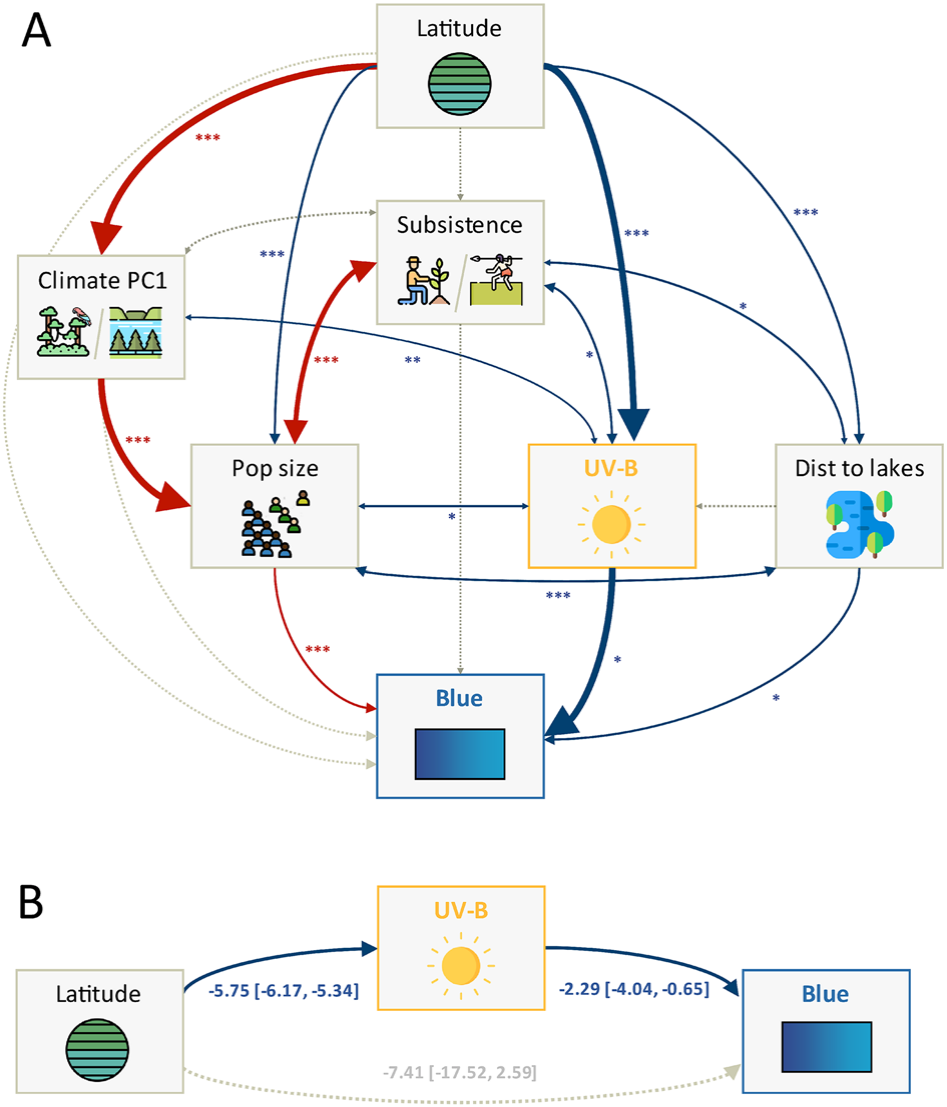
Mediation and path analyses for the presence of a distinct word for blue. (Panel **A** top): Path model with standardized coefficients (line thickness) and *p*-values (stars). Single-headed arrows represent regressions, while double-headed arrows represent covariances. Edges: solid blue = significant negative effects (at alpha-level 0.05), solid red = significant positive effects, and dotted gray = not significant. The thickness of the line indicates effect size, with a thick line indicating an effect size $>= 0.5$ and thin effect size $< 0.5$; significance stars near each arrow (*** for $p<=0.001$; ** for $p<=0.01$ and * for $p<=.0.05$). (Panel **B** bottom): Mediation analysis showing the total, direct, and indirect effects (as point estimate with 95% HDI and p-values), as well as regression coefficients (as point estimates with *p*-values in parentheses). Note, because the outcome is binary, the direct and indirect effects may be on different scales. Icons made by Freepik https://www.freepik.com from Flaticon https://www.flaticon.com/.

The mediation analysis (Fig. 2 Panel B) shows that *latitude* has a positive *total effect* (*TE* = 5.7, 95%HDI [2.3, 9.6]) on the presence of a distinct word for *blue*. There was a negative *direct effect* (*DE* = $-7.4 [-17.9, 2.4]$), as well as a positive *indirect effect* mediated by *UV-B* (*IE* = 13.0[3.6, 23.1]; $\beta _{latitude \rightarrow UVB} = -5.8 [-6.2, -5.3]$, $\beta _{UVB \rightarrow blue | latitude} = -2.3 [-4.0, -0.6]$). We explored other mediation models (see Supplementary Materials) which, taken together, suggest that *distance to lakes* also mediates between *latitude* and *blue*, and that *subsistence* is linked to *blue* through *population size*. We also constructed a complex (but largely theoretically motivated and conservative) path model (Fig. 2 Panel A) linking *latitude* and *blue* through *UV-B* and controlling for potential confounds. This model fitted the data very well ($\chi ^2(1) = 0.4$, $p = 0.53$; $CFI = 1.0$, $TLI = 1.05$, $NNFI = 1.05$, $RMSEA = 0.0$) and, importantly, showed that the effect of *latitude* on *blue* is fully mediated not only by *UV-B* ($\beta _{latitude \rightarrow UVB} = -0.96$, $p < 0.001$, and $\beta _{UVB \rightarrow blue} = -0.65$, $p = 0.032$), but also by *subsistence*, *population size* and *distance to lakes*.

**Figure 3:**
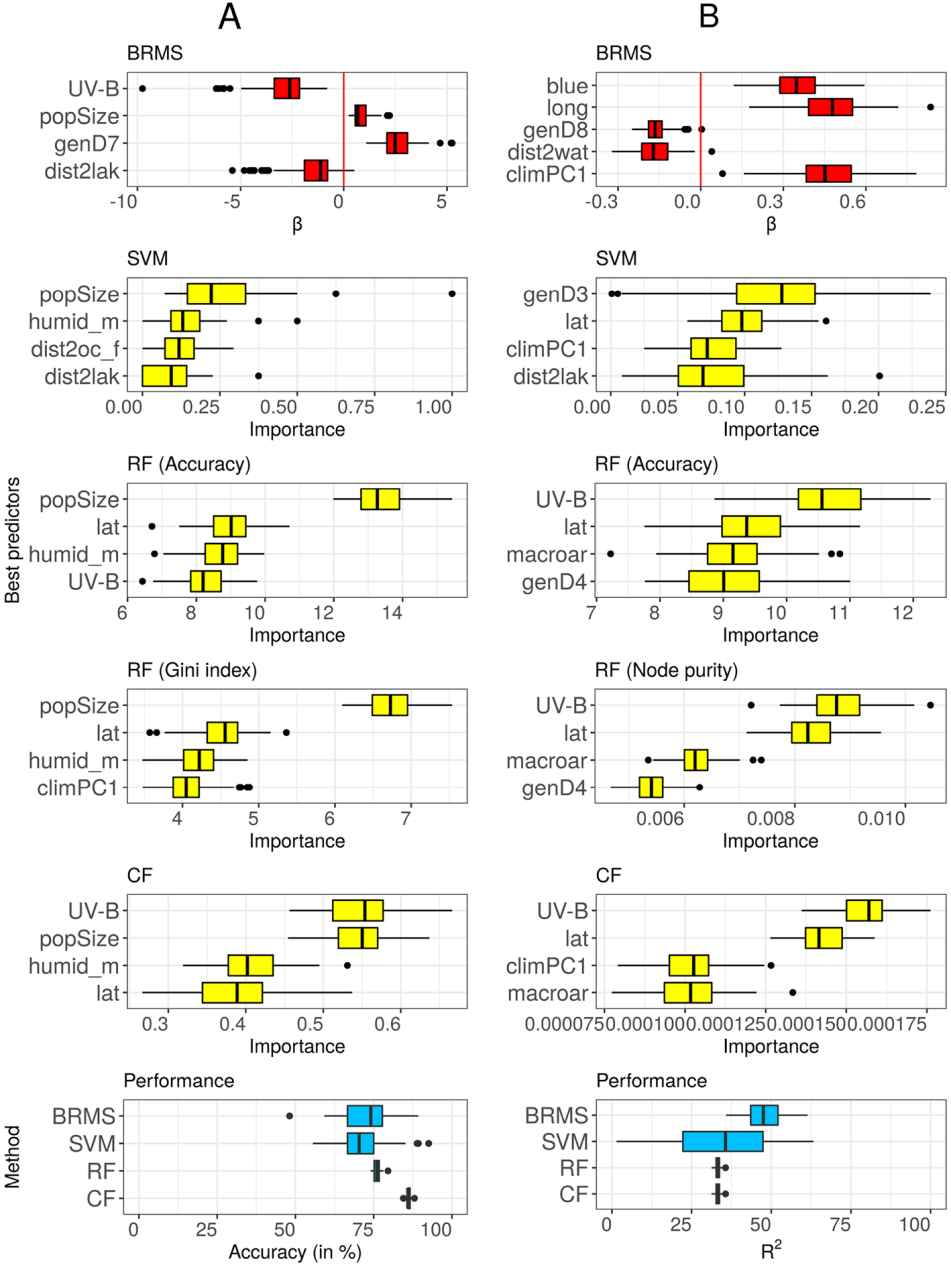
Predicting the existence of a dedicated word for blue (Panel **A**, left) and frequency of abnormal red-green color perception (Panel **B**, right); only the 7 best predictors are presented here (see Methods). 1st row: Using Bayesian mixed-effect models (BRMS), the best predictors’ (according to Bayes Factor, WAIC, LOO and K-Fold methods) slope estimates. 2nd row: specificity-based predictor importance from SVMs. 3rd row: accuracy-based predictor importance from random forests (RF), measuring the amount by which the accuracy decreases when one variable is removed from the model; higher values represent more important predictors. 4th row: Gini-index-based predictor importance from random forests (RF); this measures by how much the Gini impurity decreases when a variable is chosen to split a node (note, only relative values matter, and there is a bias towards using numeric variables to split nodes). 5th row: unconditional predictor importance from conditional random forests (CF); this is similar to the accuracy-based importance from random forests. 6th row: the performance of the four methods (BRMS, SVM, RF and CF) in terms of accuracy (left; as this is a binary classification problem) and $R^2$ (right; as this is a regression problem). Variable names have been abbreviated for legibility: *popSize* is population size, *humid_m* is median humidity, *dist2lak* is the distance to the closest lake, lat is latitude, *genD4* is the 4th dimension of the multidimensional scaling of the between-populations genetic distances, *climPC1* is the 1st principal component resulting from the Principal Component Analysis (PCA) of the climate variables, *macroar* is the macroarea, *long* is the longitude, and *dist2wat* is the distance to the closest body of water (ocean/sea, lake or river). Plots generated using R^59^.

The presence of a distinct term for *blue* was predicted by information in the full dataset using Bayesian mixed-effects logistic regression with family and macroarea as random effects, the conditional random trees, the random and conditional random forests, and the Support Vector Machines, with very little difference between them (accuracy from 76% for random forests to 91% for the Bayesian mixed-effects model). Moreover, all techniques generalized well and had comparable performance (as expected, lower on the testing sets than on the full database), the best being the conditional random forests and conditional trees. Across these, the most important predictors tended to be (not in any particular order): *UV-B, population size, climate/humidity, latitude,* and *distance to lakes* (see Fig. 3 Panel A).

### Population frequency of abnormal red-green vision

**Figure 4:**
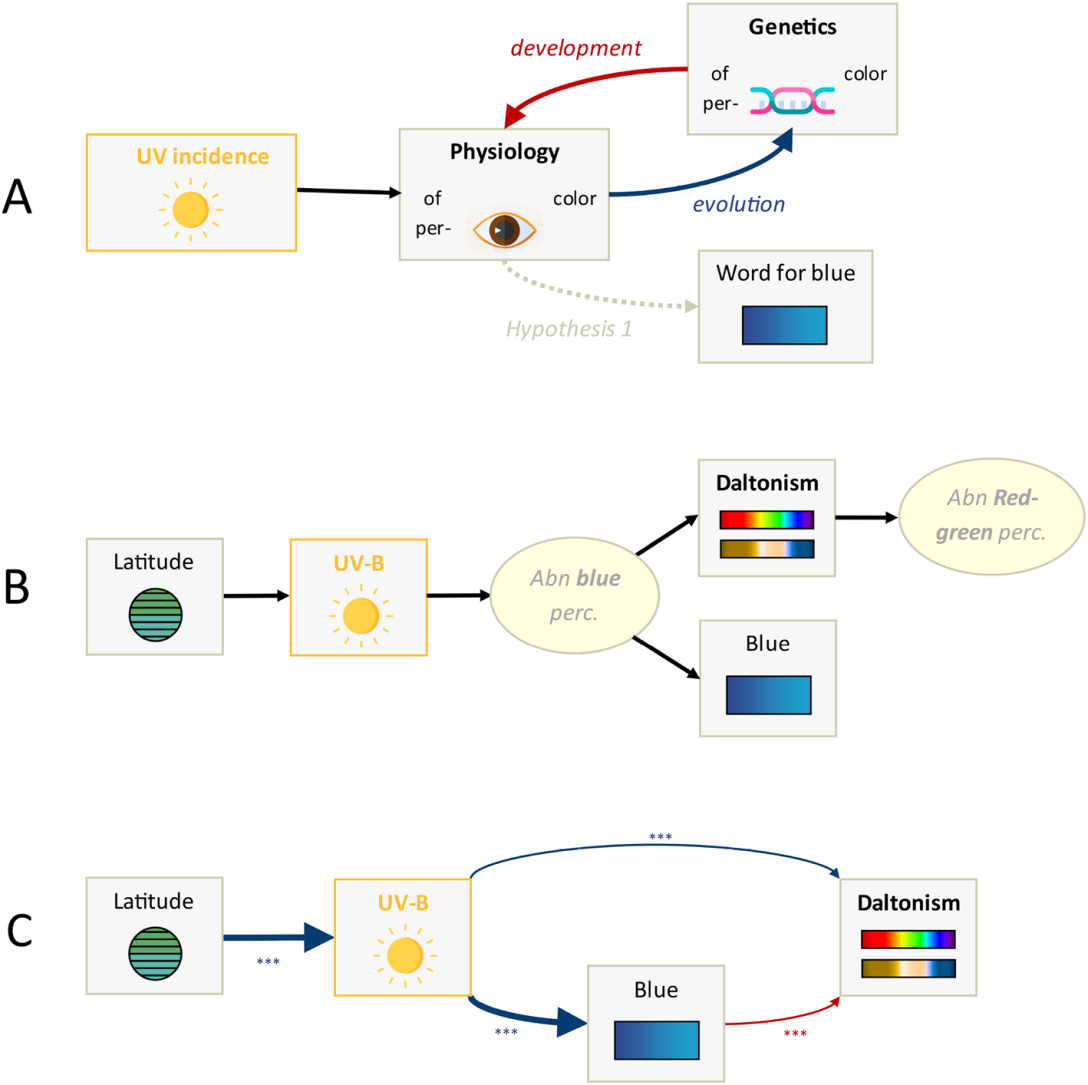
Derivation of path models for red-green abnormal color perception. (Panel ** A**): A direct graphical representation of the hypothesis that UV-B affects the unmeasured physiology of color perception during the lifetime of an individual which, in turn, affects the existence of a dedicated word for blue in the person’s language (*hypothesis 1*). In addition, the physiology of color perception also drives an evolutionary interaction with alleles resulting in abnormal red/green color perception. (Panel **B**): a Structural Equation Modelling diagram representation of Panel A with operationalized variables. (Panel **C**): The latent variable ‘abn. blue perc.’, representing the unobserved abnormal color perception of blue, is indirectly measured by two indicators (‘daltonism’ and ‘blue’), while the latent ‘abn. red-green perc.’ (abnormal red/green color perception) is not of interest here. Therefore, we used ‘blue’ as a proxy for ‘abn. blue perc.’ and ignored ‘abn. red-green perc.’, leading to the path model shown here. Icons made by Freepik https://www.freepik.com from Flaticon https://www.flaticon.com/.

In a second set of analyses, we tested whether the population frequency of abnormal red-green color perception (here, we designate this variable as *daltonism*) was linked to the incidence of UV light and the presence of a dedicated word for blue (as a proxy for its physiological effects on blue-green color perception; see Fig. 4 Panels A and B). Bayesian mixed-effects regressions showed that *daltonism* was positively influenced by *blue* ($\beta = 0.61$ [0.33, 0.88], $p(\beta =0) = 0.0004$, $p(\beta >0) = 1$) and *latitude* ($\beta = 1.69$ [0.78, 2.64], $p(\beta =0) = 0.0076$, $p(\beta <0) = 0$) , and negatively influenced by *UV-B* ($\beta = -0.3$ [− 0.45, − 0.15], $p(\beta =0) = 0.083$, $p(\beta <0) = 1$); it was also affected by 4 genetic distance dimensions (see Supplementary Materials).

Taken together, the mediation analyses suggested that the effect of *latitude* on *daltonism* was mediated mostly by *UV-B* and *blue*, and especially the mediation *UV-B* $\rightarrow$ *blue* $\rightarrow$ *daltonism* was important (*TE* = − 0.74 [− 1.21, − 0.35], *DE* = − 0.21 [− 0.35, − 0.06], *IE* = − 0.53 [− 1.04, − 0.16]; $\beta _{UVB \rightarrow blue} = -1.14$ [− 1.78, − 0.55], $\beta _{blue \rightarrow daltonism | UVB} = 0.49$ [0.20, 0.77]). A path model (Fig. 4 Panel C) fits the data well ($\chi ^{2}(1) = 4.0$, *p* = 0.137; CFI = 0.951, TLI = 0.927, NNFI = 0.927, RMSEA = 0.08) and showed that *UV-B* has both a direct effect on *daltonism* ($\beta = -0.31$, *p* < 0.001) and one mediated by *blue* ($\beta _{UVB \rightarrow blue} = -0.55$, *p* < 0.001; $\beta _{blue \rightarrow daltonism} = 0.4$, *p* < 0.001). Adding *subsistence* to the model improved the fit further ($\chi ^{2}(1) = 1.6$, *p* = 0.45; CFI = 1.000, TLI = 1.011, NNFI = 1.011, RMSEA = 0.0). It added a significant influence of *subsistence* ($\beta _{subsistence \rightarrow daltonism} = 0.38$, *p* < 0.001), but removed the influence of *blue* to *daltonism* ($\beta _{blue \rightarrow daltonism} = 0.11$, *p* = 0.32).

Converging evidence across analysis methods predicted daltonism on the full dataset, but this performance did not generalize well. Moreover, the importance of the predictors varied across methods. Nevertheless, *UV-B* tended to be among the most important predictors, along with blue and some genetic distance measures (see Fig. 3 Panel B).

## Discussion

Taken together, these analyses demonstrate that no single explanatory factor explains the color lexicon. This finding resonates with the centuries of debate on this topic with myriad variables being proposed and opposed. Our results show that multiple environmental and cultural factors interact. Specifically, we found that a language is more likely to have a dedicated word for blue when it is spoken by a larger population, which resides at higher latitudes (where the incidence of UV-B radiation is lower), and near large bodies of standing water (in particular, lakes). While number of speakers is an imperfect proxy for the unmeasured and hard-to-define variable, cultural complexity, which in turn is an indirect reflection of the use of complex dyeing techniques and colored artefacts, the environmental influences were more straightforward. For example, large lakes might not always be poster-card blue, but they certainly tend to be a salient feature and reflect the sky more often than not. Importantly, the tendency of languages spoken closer to the equator to have a distinct term for blue, is likely due to the high incidence of UV-B light, and strongly supports the proposal that acquired lens brunescence has a negative effect on the development or maintenance of a lexical distinction between ‘blue’ and ‘green’.

Overall, these results suggest that variability in color perception leads to differences in the perceptual representation of color space, which then causes differences in color lexicons. It also raises interesting questions about the mechanisms and time required for language change, given that we are considering acquired color deficiency related to aging. If language does adapt to the color vision of its speakers, then our data suggest that language adapts to the reduced capacities of older adults, despite also being spoken by younger adults and children, who are still able to perceive the distinction between green and blue. Intriguingly, other evidence suggests extreme variation in exposure to direct sunlight during very early development also affects color vision^38^. Nevertheless, our findings show a distinct developmental trajectory. Perhaps older adults change the fitness landscape to which language adapts through cultural evolution at the scale of several generations of language use and transmission^39^, a process that can be studied using agent-based computer models^40,41^. The fact that we found no influence of environmental conditions at the putative origins of language families suggests that these effects act on timescales shorter than the few thousand years usually needed for language families to differentiate. The present-day languages of the same language family descend from a single ancient proto-language and, due to migrations and language shifts on the scale of a few thousand years^42^, may end up spoken in very different locations and environments to those of their proto-language. If the color lexicon needs several thousand years to adapt to changes in UV-B incidence, then the location of these ancient proto-languages should still have a detectable effect, but the lack of such a signal in our data suggests that this change is much faster.

It is striking to note that a similar phenomenon, where language adapts to an altered fitness landscape due to acquired changes in some of the speaker population, has been proposed for several typologically striking properties of Australian Aboriginal languages and the distribution of labiodental sounds. For example, the high frequency of chronic ear infections (chronic otitis media) among Australian Aborigine children can produce partial hearing loss affecting the lower and higher parts of the auditory spectrum, resulting in a loss of fricatives, a centralized vowel system, and a long, thin consonant system in the languages of the Australian continent^43^. Similarly, consuming processed and softer foods characteristic of agriculture leads to ontogenetic changes in bite (involving the teeth and lower jaw) such that the overbite/overedge is retained into adulthood, favoring the use of labiodental sounds (such as ‘f’ and ‘v’) by languages of populations practicing agriculture^44^. Our study adds to this literature by showing that external pressures can shape semantic, as well as phonological aspects of language.

Finally, our data also supported an interaction between acquired blue-green and inherited red-green color deficits: populations closer to the equator, more affected by lens brunescence, speaking languages without a dedicated word for blue have a lower frequency of people with red-green color deficiency. The data also suggested that populations of hunter-gatherers tend to have a lower incidence of abnormal red-green color perception. While this hypothesis requires further testing, it would support the idea that certain cultural practices and subsistence strategies might have higher demands on color perception, thereby generating selective pressures against inherited color perception deficits.

Some caution is required in interpretation, however, given limitations in the current study. First, our dataset contained only 14 groups classified as hunter-gatherers, which, even if representative of the current distribution of communities, is too small to allow strong conclusions. Second, more refined genetic data—especially including information about the opsin alleles involved in red-green color deficiencies—would be necessary to better test for selection. Third, there is a need for better and, ideally, more direct measures of the exposure to complex dyeing technology and colored artefacts. Finally, populations and languages are reduced to geographic dots in our analyses, but a better approach might instead be to use aggregated measures of UV-B incidence, climate, ecology and proximity to large bodies of water across the whole area they occupy.

Nevertheless, our results strongly support the view that the color vocabulary is shaped, at least in part, by environmental factors acting on individual speakers, generating biases that are amplified by the repeated use and transmission of language in communities of similarly affected individuals. This is akin to other cases of individual biases being amplified to shape cross-linguistic diversity^45^, biases that can be either rooted in genetics^46–48^ or emerging due to environmental or cultural factors acting during the lifetime of individuals^44^.

## Methods and materials

We collected data from 142 populations, extending and re-checking earlier databases^9^ considerably, especially concerning information about the color lexicon and the incidence of abnormal red-green color perception. Each population was uniquely identified by its *Glottolog* code^49^ of the primary language spoken and its geographic location. According to *Glottolog*, these languages belong to 32 *language families* (the most represented being *Indo-European* (41), *Atlantic-Congo* (19) and *Afro-Asiatic* (13)) distributed across 6 *macroareas* (Africa (31), Australia (2), Eurasia (79), Papunesia (9), North America (9) and South America (12)). We also obtained the geographic location of the putative origins of each family^49,50^. The geographic locations were cos-transformed: cos(*longitude*) ranges between − 1.0 (− 180) and 1.0 (180), and 1.0 − cos(*latitude*) ranges between − 1.0 (the South Pole), 0.0 (the equator) and 1.0 (the North Pole).

Based on their location, for each population we obtained the following data: the list of its *geographic neighbors* (derived from the Delaunay triangulation of our geographic locations taking into account large bodies of water^51^); its log(*elevation*+1) using the Mapzen data (through R’s elevatr package); the log(*distances*) to the nearest lake, ocean, river, or large body of water in general (using the OpenStreetMap data as per^52^); and data about *specific humidity* (using data from the NOAA, as the mean of the yearly medians and interquartile ranges), and *climate* and *ecology* (as in^52^, we conducted Principal Component Analysis on the 19 variables from WorldClim for the period 1960–1990, and we retained *PC1*, explaining 49.7% of the variance and reflecting low seasonality, wet and hot climates, *PC2*, 24.7%, high seasonality, hot and dry climates, and *PC3*, 8.6%, unclear interpretation). We calculated the *incidence of ultraviolet light (UV)* using the data from NASA Total Ozone Mapping Spectrometer (TOMS) for the year 1998 (in order to replicate the procedure in^9^): these contain daily measures of UV radiation received by the human body at four wavelengths (305 nm, 310 nm, 320 nm, and 380 nm) in J/m^2^, taking into account the thickness of the ozone layer in the stratosphere, the amount of cloud cover, the elevation, and how high the sun is in the sky. Here, we use the whole-year means and standard deviations for UV-A (315 nm to 400 nm), UV-B (280 nm to 315 nm) and the full spectrum.

Moreover, we obtained each population’s log(*size*) (from^52^) and *subsistence strategy*, dichotomized into populations whose subsistence mode is primarily based on hunting, fishing, gathering and/or foraging (‘HG’) and populations with subsistence modes centered around food production (‘AGR’), using data from^44,53–55^. The information about color vocabulary specifically concerns the existence of a *specific term for blue* (a dichotomous variable, ‘no’/‘yes’), and we also collected data about the *frequency of abnormal red/green color perception* in males excluding, if specified, tritanopia, and those samples that do not distinguish between males and females, or with fewer than 50 individuals (see Fig. 1 Panel A and Supplementary Materials for details).

Finally, to control for past demography and selection (about which we do not have direct information here), we estimated the overall pairwise *genetic distances* between populations, estimated by the *fixation index* $F_{ST}$. We used a set of genetic markers aimed at forensic applications (FROG-kb) from the *ALFRED* database^56^ with ultrametric missing value imputation^57,58^, followed by classic multi-dimensional scaling (MDS), retaining the first 10 dimensions (goodness-of-fit 10.5%).

We used the following short variable names: *glottocode* (Glottolog language code), *family* (language family), *macroarea* (the macroarea), *latitude* and *longitude* (the transformed geographical coordinates), *elevation* (transformed elevation), *distance to ocean*, *distance to lakes*, *distance to rivers* and *distance to water* (transformed distances to bodies of water), *humidity median* and *humidity IQR* (humidity), *climate PC1*, *climate PC2*, *climate PC3* (the first 3 climate Principal Components, z-scored), *mean UV-A*, *sd UV-A*, *mean UV-B*, *sd UV-B*, *mean UV*, *sd UV* (UV incidence, z-scored), *population size* (transformed population size), *subsistence* (subsistence strategy), *blue* (is there a specific term for blue?), *daltonism* (frequency of abnormal red/green color perception), and *genetic D1* ... *genetic D10* (first 10 MDS dimensions of the genetic distances matrix); the suffix *_family* refers to the values at the putative family origins.

All analyses were done using R^59^. We used five main classes of methods in our analyses (see the Supplementary Materials). First, in order to understand the geographical patterning of our data, we tested it against *complete spatial randomness*^60,61^ using the the $\chi ^2$ test based on quadrat counts, and the *G*, *F* and *K* functions, and we also estimated its spatial autocorrelation using Moran’s *I* (with either the inverse of the shortest geographical distance “as the crow flies”, or the nearest neighbor distance on the Delaunay triangulation). Second, we fitted *Bayesian mixed-effect regression models* (as implemented by R’s package brms^62^ using Stan^63^) with *family* and *macroarea* as random effects, and explanatory variables and potential confounds as predictors. For *blue* we performed logistic regression, and for *daltonism* we performed beta regression (after replacing the 0.0% values by 0.01%); we used model selection based on Bayes Factors (BF), leave-one-out cross-validation (LOO), the Widely Applicable Information Criterion (WAIC), and K-Fold Cross-Validation (KFOLD, with *K* = 10). Third, we conducted *mediation analysis* using a Bayesian mixed effects model (brms with Stan) approach which allowed us to include random effects. Fourth, we used path analysis (as implemented by lavaan^64^) to test more complex models involving multiple potential predictors (but without controlling for the non-independence due to *family* and *macroarea*). Finally, we quantified the predictive capacity of a set of variables using Bayesian mixed-effects regression (brms with Stan), conditional inference trees (ctree^65^ in package partykit^66^), random forests (package randomForest^67^) and conditional random forests (cforest in package partykit) and Support Vector Machines (rminer package^68^).

## Supplementary Information

Supplementary Information.

## Data Availability

All data and scripts needed to reproduce the results reported in this paper are available in the *GitHub* repository https://github.com/ddediu/colors-UV and on *Zenodo* at 10.5281/zenodo.5083676.

## References

[1] Berlin B, Kay P (1969). Basic Color Terms: Their Universality and Evolution.

[2] Regier T, Kay P, Khetarpal N (2007). Color naming reflects optimal partitions of color space. Proc. Natl. Acad. Sci..

[3] Gibson E (2017). Color naming across languages reflects color use. Proc. Natl. Acad. Sci..

[4] Zaslavsky N, Kemp C, Tishby N, Regier T (2019). Color naming reflects both perceptual structure and communicative need. Top. Cogn. Sci..

[5] Conway BR, Ratnasingam S, Jara-Ettinger J, Futrell R, Gibson E (2020). Communication efficiency of color naming across languages provides a new framework for the evolution of color terms. Cognition.

[6] Malt BC, Majid A (2013). How thought is mapped into words. Wiley Interdiscip. Rev. Cognit. Sci..

[7] Wierzbicka A (1990). The meaning of color terms: Semantics, culture, and cognition. Cognit. Linguist..

[8] Rivers WHR (1901). Primitive Color Vision.

[9] Brown, A. M. & Lindsey, D. T. Color and language: Worldwide distribution of Daltonism and distinct words for “blue”. *Visual Neurosci.* **21**, 409–412. 10.1017/S0952523804213098 (2004). 10.1017/s0952523804213098 15518222

[10] Webster MA, Mizokami Y, Webster SM (2007). Seasonal variations in the color statistics of natural images. Network.

[11] Conklin HC (1973). Color categorization. Am. Anthropol..

[12] Levinson SC (2000). Yeli Dnye and the theory of basic color terms. J. Linguist. Anthropol..

[13] Naroll R (1970). What have we learned from cross-cultural surveys?. Am. Anthropol..

[14] Ember M (1978). Size of color lexicon: Interaction of cultural and biological factors. Am. Anthropol..

[15] Bornstein MH (1973). Color vision and color naming: A psychophysiological hypothesis of cultural difference. Psychol. Bull..

[16] Lindsey DT, Brown AM (2002). Color naming and the phototoxic effects of sunlight on the eye. Psychol. Sci..

[17] Hammond BR (2012). The visual effects of intraocular colored filters. Scientifica.

[18] Remington LA (2012). Clinical Anatomy and Physiology of the Visual System.

[19] Javitt JC, Taylor HR (1995). Cataract and latitude. Doc. Ophthalmol..

[20] Werner JS, Peterzell DH, Scheetz AJ (1990). Light, vision, and aging. Optomet. Vis. Sci..

[21] Young RW (1990). Age-Related Cataract.

[22] Pokorny J, Smith VC, Lutze M (1987). Aging of the human lens. Appl. Opt..

[23] Hightower KR (1995). The role of the lens epithelium in development of UV cataract. Curr. Eye Res..

[24] West SK (1998). Sunlight exposure and risk of lens opacities in a population-based study: The salisbury eye evaluation project. JAMA.

[25] Young RW (1994). The family of sunlight-related eye diseases. Optom. Vis. Sci..

[26] Werner, J. S., Schefrin, B. E. & Bradley, A. Optics and vision of the aging eye. In *Handbook of Optics*, Vol. 3, 20–30 (McGraw-Hill, 2010).

[27] Delahunt PB, Webster MA, Ma L, Werner JS (2004). Long-term renormalization of chromatic mechanisms following cataract surgery. Vis. Neurosci..

[28] Tregillus, K. E. M., Werner, J. S. & Webster, M. A. Adjusting to a sudden “aging” of the lens. *J. Opt. Soc. Am. A* **33**, 129. 10.1364/JOSAA.33.00A129 (2016). 10.1364/JOSAA.33.00A129 PMC4765957 26924924

[29] Weale RA (1988). Age and the transmittance of the human crystalline lens. J. Physiol..

[30] Walter, S. Perceiving “grue”: filter simulations of aged lenses support the lens-brunescence hypothesis and reveal individual categorization types. In Biggam, C. P., Hough, C., Kay, C. & Simmons, D. R. (eds.) *New Directions in Colour Studies*, 329–342. 10.1075/z.167.37wal (John Benjamins Publishing Company, 2011).

[31] Ladd DR, Roberts SG, Dediu D (2015). Correlational studies in typological and historical linguistics. Ann. Rev. Linguist..

[32] Henrich J (2004). Demography and cultural evolution: How adaptive cultural processes can produce maladaptive losses: The Tasmanian case. Am. Antiq..

[33] Powell A, Shennan S, Thomas MG (2009). Late Pleistocene demography and the appearance of modern human behavior. Science.

[34] Stark AE (2020). On random and systematic variation in the prevalence of defective color vision. Twin Res. Hum. Genet..

[35] Adam A (1969). A further query on color blindness and natural selection. Soc. Biol..

[36] McElreath, R. *Statistical Rethinking: A Bayesian Course with Examples in R and Stan*. CRC Texts in Statistical Science, 2 edn. (Taylor and Francis, 2020).

[37] Holsinger KE, Weir BS (2009). Genetics in geographically structured populations: Defining, estimating and interpreting FST. Nat. Rev. Genet..

[38] Laeng B (2007). Latitude-of-birth and season-of-birth effects on human color vision in the Arctic. Vision. Res..

[39] Richerson, P. J. & Christiansen, M. H. (eds) *Cultural Evolution: Society, Technology, Language, and Religion* (The MIT Press, 2013).

[40] Jameson KA, Komarova NL (2009). Evolutionary models of color categorization. I. Population categorization systems based on normal and dichromat observers. J. Opt. Soc. Am. A.

[41] Jameson KA, Komarova NL (2009). Evolutionary models of color categorization. II. Realistic observer models and population heterogeneity. J. Opt. Soc. Am. Opt. Image Sci. Vis..

[42] Bowern, C. & Evans, B. *The Routledge Handbook of Historical Linguistics*, 1 edn. (Routledge, 2014).

[43] Butcher, A. Australian aboriginal languages: Consonant-salient phonologies and the ‘Place-of-Articulation Imperative’. In Harrington, J. & Tabain, M. (eds.) *Speech Production: Models, Phonetic Processes, and Techniques, Macquarie Monographs in Cognitive Science*, 187–210 (Psychology Press, 2006).

[44] Blasi DE (2019). Human sound systems are shaped by post-Neolithic changes in bite configuration. Science.

[45] Dediu D, Janssen R, Moisik SR (2017). Language is not isolated from its wider environment: Vocal tract influences on the evolution of speech and language. Lang. Commun..

[46] Dediu D, Ladd DR (2007). Linguistic tone is related to the population frequency of the adaptive haplogroups of two brain size genes, ASPM and Microcephalin. Proc. Natl. Acad. Sci..

[47] Dediu D, Janssen R, Moisik SR (2019). Weak biases emerging from vocal tract anatomy shape the repeated transmission of vowels. Nat. Hum. Behav..

[48] Moisik SR, Dediu D (2017). Anatomical biasing and clicks: Evidence from biomechanical modeling. J. Lang. Evol..

[49] Hammarström, H., Bank, S., Forkel, R. & Haspelmath, M. *Glottolog 3.2* (Max Planck Institute for the Science of Human History, 2018).

[50] Wichmann, S., Müller, A. & Velupillai, V. Homelands of the world’s language families: A quantitative approach. *Diachronica* **27**, 247–276. 10.1075/dia.27.2.05wic (2010).

[51] Cysouw, M., Dediu, D. & Moran, S. Comment on “Phonemic diversity supports a serial founder effect model of language expansion from Africa”. *Science* **335**, 657–657. 10.1126/science.1208841 (2012). 10.1126/science.1208841 22323802

[52] Bentz C, Dediu D, Verkerk A, Jäger G (2018). The evolution of language families is shaped by the environment beyond neutral drift. Nat. Hum. Behav..

[53] Bickel, B. *et al.* The AUTOTYP typological databases (Version 0.1.0) (2017).

[54] Kirby K (2016). D-PLACE: A global database of cultural, linguistic and environmental diversity. PLoS ONE.

[55] Turchin, P. *et al.* Seshat: The global history databank. *Cliodynamics*. 10.21237/C7clio6127917 (2015).

[56] Rajeevan H (2003). ALFRED: The ALelle FREquency database. Nucleic Acids Res..

[57] De Soete G (1984). Ultrametric tree representations of incomplete dissimilarity data. J. Classif..

[58] Lapointe F, Kirsch J (1995). Estimating phylogenies from lacunose distance matrices, with special reference to DNA hybridization data. Mol. Biol. Evol..

[59] R Core Team. *R: A Language and Environment for Statistical Computing* (R Foundation for Statistical Computing, 2021).

[60] Baddeley, A., Rubak, E. & Turner, R. *Spatial Point Patterns: Methodology and Applications with R*. Champan & Hall/CRC Interdisciplinary Statistics Series (CRC Press, 2016).

[61] Spielman, S. E. Point pattern analysis. In *International Encyclopedia of Geography*, 1–9, 10.1002/9781118786352.wbieg0849 (American Cancer Society, 2017).

[62] Bürkner P-C (2018). Advanced Bayesian multilevel modeling with the R package brms. R J..

[63] Guo, J. *et al.* rstan: R Interface to Stan (2020).

[64] Rosseel Y (2012). lavaan: An R package for structural equation modeling. J. Stat. Softw..

[65] Hothorn T, Hornik K, Zeileis A (2006). Unbiased recursive partitioning: A conditional inference framework. J. Comput. Graph. Stat..

[66] Hothorn T, Zeileis A (2015). partykit: A modular toolkit for recursive partytioning in R. J. Mach. Learn. Res..

[67] Liaw, A. & Wiener, M. Classification and regression by RandomForest. *Forest* **23**, (2001).

[68] Cortez, P. Data mining with neural networks and support vector machines using the R/rminer Tool. In Perner, P. (ed.) *Advances in Data Mining. Applications and Theoretical Aspects, Lecture Notes in Computer Science*, 572–583, 10.1007/978-3-642-14400-4_44 (Springer, 2010).

