# Supplementary material for: Environment and culture shape both the colour lexicon and the genetics of colour perception
Source: Sci Rep. 2021 Sep 27;11:19095. doi: 10.1038/s41598-021-98550-3 (PMC8476573; doi:10.1038/s41598-021-98550-3)
Supplement: Supplementary file 1 — Supplementary Information. [file 41598_2021_98550_MOESM1_ESM.html]

Environment and culture shape both the colour lexicon and the genetics of colour perception


# Environment and culture shape both the colour lexicon and the genetics of colour perception

### Supporting information: full analyses and plots

#### Mathilde Josserand [mathilde.josserand@gmail.fr], Emma Meeussen, Asifa Majid, and Dan Dediu [ddediu@gmail.com]

#### Mon Jul 19 12:51:52 2021

# 1 Introduction

This `HTML` document (possibly, printed to `PDF`) resulted from compiling the corresponding `Rmarkdown` script and contains all the results and plots supporting the paper *Environment and culture shape both the colour lexicon and the genetics of colour perception*. All the data and scripts needed to reproduce this document are available in the *GitHub* repository https://github.com/ddediu/colors-UV and on *Zenodo* at doi:10.5281/zenodo.5083676.

## 1.1 Typographic conventions

This `HTML` document uses the following font and color conventions:

- *regular text* is rendered as “regular text”;
- *emphasis* is represented using *italic text*, **bold text** or, in rare circumstances, ***bold+italic text***;
- *software* or programming concepts (e.g., applications, packages or function names) are represented using `fixed font text`;
- *section heads* use specific font sizes and are numbered;
- *hyperlinks* to sections of this document and to external resources on the web are represented as link to the Introduction or link to R project’s website, and can be clicked to navigate there;
- *notes* are represented as numbered superscripts1 which can be clicked to go to the note’s text;
- *captions* use **bold font**, are numbered, and are placed below the corresponding figure and above the corresponding table;
- *raw output*, as produced by various `R` functions and expressions (for example, as in the Appendices), is shown using `fixed font text` in clearly marked boxes;
- the *floating document outline* on the left shows the main sections and the path to the current location and is clickable for quick navigation.

Please note that these font and color conventions can be changed in the `Rmarkdown` script (for example, selecting a different theme in `RStudio`); here, we used *cerulean* and *textmate*.

## 1.2 Software and hardware info

The full information about the version of `R` (R Core Team, 2021), the packages and the hardware and software platform used to obtain this document are given in the Section *Session information* at the end of this document2. The figures were made using various `R` packages; in particular, the maps were drawn using the `maps` package (wrapped by the `map_data()` function in `ggplot2`), which, in turn uses public domain data from the Natural Earth project.

# 2 Data

This dataset is built starting from the data accompanying Brown & Lindsey (2004), to which Emma Meeussen added several new populations (and checked the pre-existing coding) using a variety of primary and secondary sources (see Meeussen (2015) for details), supplemented with several environmental variables by Mathilde Josserand (see Josserand (2020)).

Thus, it is important to note that the databases (the one accompanying Brown & Lindsey (2004) and ours) are similar but they are not exact copies. Firstly, our dataset contains more languages (142 compared to 118). Although it may look like an extension of the prior research, our database was not a superset of the earlier database. In fact, in some cases where the same source was used, different incidence percentages were chosen as more informative or our judgement differed from the authors’ with respect to linguistic or ethnologic classification, resulting in slight differences between the databases (Meeussen, 2015).

## 2.1 Populations and languages

In our dataset, there are 142 populations, each uniquely identified by the Glottolog code (the *glottocode*) of the primary language they speak (the matching was done manually).

## 2.2 Geographic location

The geographic coordinates of the populations were retrieved from the Glottolog based on their *glottocode*s. As per Brown & Lindsey (2004), we computed the cosine of these *latitude*s and the *longitude*s to be used in the statistical models; while `cos(latitude)` captures the closeness to the equator and ranging between 0.0 (one of the poles) and 1.0 (the equator), we use here `1.0 - cos(latitude)` so that they are 0.0 at the equator and 1.0 at the poles, converting the “natural” interpretation of latitudes, and `cos(longitude)`range between -1.0 and 1.0 (corresponding to -180 and 180 degrees, respectively); please note that longitude can be coded (and plotted) either as ranging between 0° and 360°, or between -180° and +180°, when we need to avoid the International Date Line (IDL) producing an artefactual boundary.

In the following plot, we plot exactly the same information but using a different format:

**Figure 1.** Map of the populations in our sample (with language names and details for Europe and the Indian subcontinent). Figure generated using `R` version 4.0.5 (2021-03-31) and packages `ggplot2` (version 3.3.5), `maps` (version 3.3.0). Maps are using public domain data from the Natural Earth project as provided by the `R` package `maps`.

**Figure 2.** Relationship between raw and transformed values for latitude (left, blue) and longitude (right, red); please note that, as opposed to the map above, longitude was shifted so that longitudes > 180° are negative (i.e., centered on 0°). Figure generated using `R` version 4.0.5 (2021-03-31) and package `ggplot2` (version 3.3.5).

## 2.3 Elevation

We obtained elevation (altitude, in meters) using Mapzen data, which is still available (July 2020) on the Terrain Tiles on Amazon Web Services and accessible through the `elevatr` package; please note that for the analyses, we use `log(elevation + 1)` (we added 1m to avoid errors for the locations recorded at sea level, 0m).

| Min. | 1st Qu. | Median | Mean | 3rd Qu. | Max. |
| --- | --- | --- | --- | --- | --- |
| 0 | 118.1 | 303 | 624.7 | 916.6 | 4164 |

**Figure 3.** Distribution of elevation. Figure generated using `R` version 4.0.5 (2021-03-31) and package `ggplot2` (version 3.3.5).

**Figure 4.** Map of elevation (color scale). Figure generated using `R` version 4.0.5 (2021-03-31) and package `ggplot2` (version 3.3.5). Maps are using public domain data from the Natural Earth project as provided by the `R` package `maps`.

**Figure 5.** Relationship between raw and transformed values for elevation. Figure generated using `R` version 4.0.5 (2021-03-31) and package `ggplot2` (version 3.3.5).

## 2.4 Climate and ecology

We reused the code from Bentz, Dediu, Verkerk, & Jäger (2018) to extract historical data on global weather and climate from WorldClim for the period 1960-1990, encoded in 19 variables covering various measures (such as temperature, seasonality or precipitation). As in Bentz, Dediu, Verkerk, & Jäger (2018), we conduced a A Principal Component Analysis (PCA) for our data only, and we found similar results, namely that the first two principal components (PCs) explain most of the data and have meaningful interpretations: the PC1 explains 49.7% of the variance and reflects low seasonality, wet and hot climate, whereas PC2 explains 24.7% of the variance and reflects high seasonality, hot and dry climate; PC3 explains only 8.6% and its interpretation is less straightforward; see Bentz, Dediu, Verkerk, & Jäger (2018) for details (please note that the sign of the PCs is arbitrary). For the analyses, these variables were z-scored (thus, the relationship between raw and transformed values is linear).

### 2.4.1 Climate PC1

| Min. | 1st Qu. | Median | Mean | 3rd Qu. | Max. |
| --- | --- | --- | --- | --- | --- |
| -6.449 | -2.016 | -0.06516 | 0.1165 | 2.43 | 7.14 |

**Figure 6.** Distribution of climate PC1. Figure generated using `R` version 4.0.5 (2021-03-31) and package `ggplot2` (version 3.3.5).

**Figure 7.** Map of climate PC1 (color scale). Figure generated using `R` version 4.0.5 (2021-03-31) and packages `ggplot2` (version 3.3.5), `maps` (version 3.3.0). Maps are using public domain data from the Natural Earth project as provided by the `R` package `maps`.

### 2.4.2 Climate PC2

| Min. | 1st Qu. | Median | Mean | 3rd Qu. | Max. |
| --- | --- | --- | --- | --- | --- |
| -5.762 | -1.313 | -0.02372 | 0.07512 | 1.458 | 4.841 |

**Figure 8.** Distribution of climate PC2. Figure generated using `R` version 4.0.5 (2021-03-31) and package `ggplot2` (version 3.3.5).

**Figure 9.** Map of climate PC2 (color scale). Figure generated using `R` version 4.0.5 (2021-03-31) and packages `ggplot2` (version 3.3.5), `maps` (version 3.3.0). Maps are using public domain data from the Natural Earth project as provided by the `R` package `maps`.

### 2.4.3 Climate PC3

| Min. | 1st Qu. | Median | Mean | 3rd Qu. | Max. |
| --- | --- | --- | --- | --- | --- |
| -3.886 | -0.5746 | 0.1997 | 0.05937 | 0.9958 | 2.144 |

**Figure 10.** Distribution of climate PC3. Figure generated using `R` version 4.0.5 (2021-03-31) and package `ggplot2` (version 3.3.5).

**Figure 11.** Map of climate PC3 (color scale). Figure generated using `R` version 4.0.5 (2021-03-31) and packages `ggplot2` (version 3.3.5), `maps` (version 3.3.0). Maps are using public domain data from the Natural Earth project as provided by the `R` package `maps`.

## 2.5 Humidity

We obtained specific humidity (“the mass of water vapour in a unit mass of moist air, usually expressed as grams of vapour per kilogram of air”; Encyclopaedia Britannica) data from the NOAA, given as monthly extractions between 1 January 1949 and the download date, and we computed the overall mean, median, standard deviation and IQR across all measurements, as well as the mean across the yearly means, medians, standard deviations and IQRs. However, all these measures are highly correlated by type (see plots below), such that we only retain here the mean of the yearly medians and IQRs. We did not transform these variables for the analyses.

**Figure 12.** Pairwise correlations between measures of central tendency for humidity. Figure generated using `R` version 4.0.5 (2021-03-31)

**Figure 13.** Pairwise correlations between measures of dispersion for humidity. Figure generated using `R` version 4.0.5 (2021-03-31)

### 2.5.1 Median (mean of yearly medians)

| Min. | 1st Qu. | Median | Mean | 3rd Qu. | Max. |
| --- | --- | --- | --- | --- | --- |
| 0.002379 | 0.006159 | 0.009011 | 0.01038 | 0.01461 | 0.01953 |

**Figure 14.** Distribution of median humidity. Figure generated using `R` version 4.0.5 (2021-03-31) and package `ggplot2` (version 3.3.5).

**Figure 15.** Map of median humidity (color scale). Figure generated using `R` version 4.0.5 (2021-03-31) and packages `ggplot2` (version 3.3.5), `maps` (version 3.3.0). Maps are using public domain data from the Natural Earth project as provided by the `R` package `maps`.

### 2.5.2 Variation (mean of yearly IQRs)

| Min. | 1st Qu. | Median | Mean | 3rd Qu. | Max. |
| --- | --- | --- | --- | --- | --- |
| 0.000513 | 0.00205 | 0.004153 | 0.004522 | 0.00597 | 0.0121 |

**Figure 16.** Distribution of variation in humidity (IQR). Figure generated using `R` version 4.0.5 (2021-03-31) and package `ggplot2` (version 3.3.5).

**Figure 17.** Map of variation in humidity (IQR) (color scale). Figure generated using `R` version 4.0.5 (2021-03-31) and packages `ggplot2` (version 3.3.5), `maps` (version 3.3.0). Maps are using public domain data from the Natural Earth project as provided by the `R` package `maps`.

## 2.6 Distance to bodies of water

We reused the code from Bentz, Dediu, Verkerk, & Jäger (2018) to compute the distances from each of our populations to the nearest lake, ocean, river, and water in general, using OpenStreetMap raster files3. For the analyses, we use the `log` of these distances.

**Figure 18.** Relationship between raw and transformed values for distance to large bodies of water. For left to right and top to bottom, distances to: lakes, rivers, oceans and water in general. Figure generated using `R` version 4.0.5 (2021-03-31) and package `ggplot2` (version 3.3.5).

### 2.6.1 Distance to lakes

| Min. | 1st Qu. | Median | Mean | 3rd Qu. | Max. |
| --- | --- | --- | --- | --- | --- |
| 0.6365 | 4.932 | 13.52 | 28.38 | 28.88 | 295 |

**Figure 19.** Distribution of distances to lakes. Figure generated using `R` version 4.0.5 (2021-03-31) and package `ggplot2` (version 3.3.5).

**Figure 20.** Map of distances to lakes (color scale). Figure generated using `R` version 4.0.5 (2021-03-31) and packages `ggplot2` (version 3.3.5), `maps` (version 3.3.0). Maps are using public domain data from the Natural Earth project as provided by the `R` package `maps`.

### 2.6.2 Distance to rivers

| Min. | 1st Qu. | Median | Mean | 3rd Qu. | Max. |
| --- | --- | --- | --- | --- | --- |
| 1.29 | 12.72 | 31.27 | 80.73 | 59.07 | 1816 |

**Figure 21.** Distribution of distances to rivers. Figure generated using `R` version 4.0.5 (2021-03-31) and package `ggplot2` (version 3.3.5).

**Figure 22.** Map of distances to rivers (color scale). Figure generated using `R` version 4.0.5 (2021-03-31) and packages `ggplot2` (version 3.3.5), `maps` (version 3.3.0). Maps are using public domain data from the Natural Earth project as provided by the `R` package `maps`.

### 2.6.3 Distance to oceans

| Min. | 1st Qu. | Median | Mean | 3rd Qu. | Max. |
| --- | --- | --- | --- | --- | --- |
| 3.419 | 54.98 | 169.6 | 321.3 | 519.2 | 1217 |

**Figure 23.** Distribution of distances to oceans. Figure generated using `R` version 4.0.5 (2021-03-31) and package `ggplot2` (version 3.3.5).

**Figure 24.** Map of distances to oceans (color scale). Figure generated using `R` version 4.0.5 (2021-03-31) and packages `ggplot2` (version 3.3.5), `maps` (version 3.3.0). Maps are using public domain data from the Natural Earth project as provided by the `R` package `maps`.

### 2.6.4 Distance to water

| Min. | 1st Qu. | Median | Mean | 3rd Qu. | Max. |
| --- | --- | --- | --- | --- | --- |
| 0.6365 | 4.613 | 9.37 | 19.34 | 24.08 | 238.6 |

**Figure 25.** Distribution of distances to water. Figure generated using `R` version 4.0.5 (2021-03-31) and package `ggplot2` (version 3.3.5).

**Figure 26.** Map of distances to water (color scale). Figure generated using `R` version 4.0.5 (2021-03-31) and packages `ggplot2` (version 3.3.5), `maps` (version 3.3.0). Maps are using public domain data from the Natural Earth project as provided by the `R` package `maps`.

## 2.7 UV incidence

The incidence of ultra-violet light (UV) was calculated from the data available from the NASA Total Ozone Mapping Spectrometer (TOMS)4 for the year 1998, in order to replicate the procedure in Brown & Lindsey (2004). These data contain daily measures of UV radiation received by the human body at four wavelengths (305 nm, 310 nm, 320 nm, and 380 nm) in Joules per square meter (J/m2), taking into account the thickness of the ozone layer in the stratosphere, the amount of cloud cover, the elevation, and how high the sun is in the sky. Here, we computed the mean and standard deviation for the whole year for UV-A (315 nm to 400 nm), for UV-B (280 nm to 315 nm) and for the full spectrum; these were further z-scored for the statistical analyses (thus, the relationship between raw and transformed values is linear).

### 2.7.1 UV-A

Summaries for mean and standard deviation:

| Min. | 1st Qu. | Median | Mean | 3rd Qu. | Max. |
| --- | --- | --- | --- | --- | --- |
| 147 | 324.3 | 512.6 | 450.2 | 566.1 | 624 |

| Min. | 1st Qu. | Median | Mean | 3rd Qu. | Max. |
| --- | --- | --- | --- | --- | --- |
| 132 | 179.5 | 195.8 | 192 | 209.3 | 229.1 |

**Figure 27.** UV-A (mean and standard deviation). Figure generated using `R` version 4.0.5 (2021-03-31) and package `ggplot2` (version 3.3.5).

**Figure 28.** Map of UV-A (mean and standard deviation). Figure generated using `R` version 4.0.5 (2021-03-31) and packages `ggplot2` (version 3.3.5), `maps` (version 3.3.0). Maps are using public domain data from the Natural Earth project as provided by the `R` package `maps`.

### 2.7.2 UV-B

Summaries for mean and standard deviation:

| Min. | 1st Qu. | Median | Mean | 3rd Qu. | Max. |
| --- | --- | --- | --- | --- | --- |
| 10.06 | 36.66 | 84.91 | 71.03 | 101 | 117.9 |

| Min. | 1st Qu. | Median | Mean | 3rd Qu. | Max. |
| --- | --- | --- | --- | --- | --- |
| 13.35 | 26.36 | 29.67 | 28.43 | 31.6 | 40.16 |

**Figure 29.** UV-B (mean and standard deviation). Figure generated using `R` version 4.0.5 (2021-03-31) and package `ggplot2` (version 3.3.5).

**Figure 30.** Map of UV-B (mean and standard deviation). Figure generated using `R` version 4.0.5 (2021-03-31) and packages `ggplot2` (version 3.3.5), `maps` (version 3.3.0). Maps are using public domain data from the Natural Earth project as provided by the `R` package `maps`.

### 2.7.3 UV (overall)

Summaries for mean and standard deviation:

| Min. | 1st Qu. | Median | Mean | 3rd Qu. | Max. |
| --- | --- | --- | --- | --- | --- |
| 78.83 | 180.5 | 298.5 | 260.6 | 331.2 | 370.9 |

| Min. | 1st Qu. | Median | Mean | 3rd Qu. | Max. |
| --- | --- | --- | --- | --- | --- |
| 117.1 | 195.9 | 260.3 | 235.4 | 275.1 | 288.4 |

**Figure 31.** UV (mean and standard deviation). Figure generated using `R` version 4.0.5 (2021-03-31) and package `ggplot2` (version 3.3.5).

**Figure 32.** Map of UV (mean and standard deviation). Figure generated using `R` version 4.0.5 (2021-03-31) and packages `ggplot2` (version 3.3.5), `maps` (version 3.3.0). Maps are using public domain data from the Natural Earth project as provided by the `R` package `maps`.

## 2.8 Log of population size

The log of population size was obtained from Bentz, Dediu, Verkerk, & Jäger (2018).

| Min. | 1st Qu. | Median | Mean | 3rd Qu. | Max. |
| --- | --- | --- | --- | --- | --- |
| 0 | 11.01 | 14.46 | 13.78 | 16.73 | 24.25 |

**Figure 33.** Log population size. Figure generated using `R` version 4.0.5 (2021-03-31) and package `ggplot2` (version 3.3.5).

**Figure 34.** Map of log population size. Figure generated using `R` version 4.0.5 (2021-03-31) and packages `ggplot2` (version 3.3.5), `maps` (version 3.3.0). Maps are using public domain data from the Natural Earth project as provided by the `R` package `maps`.

## 2.9 Subsistence strategy

Subsistence strategy was obtained mainly from AUTOTYP (Bickel et al., 2017) as coded in (Blasi et al., 2019), supplemented with information from other databases such as D-Place (Kirby et al., 2016) and Seshat (Turchin et al., 2015). This is represented by the binary variable *subsistence* with values ‘HG’ for populations whose subsistence mode is based on hunting, fishing, gathering and/or foraging, and ‘AGR’ for populations with subsistence modes centered around food production.

| HG | AGR |
| --- | --- |
| 14 | 128 |

**Figure 35.** Subsistence strategy. Figure generated using `R` version 4.0.5 (2021-03-31) and package `ggplot2` (version 3.3.5).

**Figure 36.** Map of subsistence strategy. Figure generated using `R` version 4.0.5 (2021-03-31) and packages `ggplot2` (version 3.3.5), `maps` (version 3.3.0). Maps are using public domain data from the Natural Earth project as provided by the `R` package `maps`.

## 2.10 Color vocabulary

Brown & Lindsey (2004) and Meeussen (2015) have collected information about the color vocabularies according to the Basic Color Categories of Berlin & Kay (1991). Here, we used only one variable, namely the *presence of a specific term for blue* (variable *exists\_blue* with values “yes” or “no”).

For more precise information on the way information about the color vocabularies have been collected, please refer to the following paragraph, extracted from Meeussen (2015). If no detailed linguistic information was available on the language under investigation, an informed decision was made based on available geographical and ethnic cues in the original source combined with language maps from Ethnologue (Lewis, 2014). For each entry in the database, the corresponding native language (L1) and its ISO 639-3 three letter language identifier, obtained from Ethnologue, were added. If applicable, a second language (L2) was also included. In an extension of the database, each language was recorded in a seperate entry, which included name, ISO code, and a variable indicating whether the language had a seperate term for ‘blue.’ In order to collect the colour terms per language, dictionary data for each language were obtained from written or online dictionaries, online word lists and the Brown & Lindsey (2004) database. In case no such list or dictionary was available, dictionary data from the second language or a closely related language were used. In two cases, data from a protolanguage were used. The linguistic resources can be found in Appendix A in Meeussen (2015).

### 2.10.1 Specific term for ‘blue’

| no | yes |
| --- | --- |
| 60 | 82 |

**Figure 37.** Is there a specific term for ‘blue?’ Figure generated using `R` version 4.0.5 (2021-03-31) and package `ggplot2` (version 3.3.5).

**Figure 38.** Map of specific terms for ‘blue.’ Figure generated using `R` version 4.0.5 (2021-03-31) and packages `ggplot2` (version 3.3.5), `maps` (version 3.3.0). Maps are using public domain data from the Natural Earth project as provided by the `R` package `maps`.

## 2.11 Abnormal red/green color perception

Data on the incidence of abnormal red/green color perception for males was collected from 85 references, using the Ishihara test (Ishihara, 1917), the anomaloscope (Patent No. US3382025A, 1968), the Holmgren-Thomson wool test (Thomson, 1880), or the Hardy-Rand-Rittler pseudoisochromatic plate test (Hardy, Rand, & Rittler, 1954); see Meeussen (2015) and Josserand (2020) for details. These data represent the percent of red/green “color blind” males in the population, and, for our data, varies between 0% and 11%. Overall “color blindness” rates were used, unless specific information was available; we include only *deuteranopia*, *deuteranomaly*, *protanopia* and *protanomaly*, and we specifically did not include data on *tritanopia* (as this concerns abnormal color perception in the yellow/blue range). We excluded from the analyses samples which did not distinguish between male and female, or which had data for less than 50 individuals. We named here this variable *daltonism*, but, while this is a very short and evocative variable name, as discussed above, we emphatically also considered milder forms of red/green abnormal color perception. For some statistical analyses that do not tolerate 0s (beta regression), we replaced the reported 0% by a very small percentage (0.0001%).

The complete list of references used to obtain these data can be found in Appendix B in Meeussen (2015).

| Min. | 1st Qu. | Median | Mean | 3rd Qu. | Max. |
| --- | --- | --- | --- | --- | --- |
| 0 | 1.87 | 3.27 | 3.88 | 5.655 | 10.68 |

**Figure 39.** Incidence of red/green abnormal color perception. Figure generated using `R` version 4.0.5 (2021-03-31) and package `ggplot2` (version 3.3.5).

**Figure 40.** Map of incidence of red/green abnormal color perception (color scale). Figure generated using `R` version 4.0.5 (2021-03-31) and packages `ggplot2` (version 3.3.5), `maps` (version 3.3.0). Maps are using public domain data from the Natural Earth project as provided by the `R` package `maps`.

## 2.12 Language family

For each language, we obtained its family affiliation from the Glottolog (Hammarström, Bank, Forkel, & Haspelmath, 2018); there are 32 unique language families, most languages belonging to the Indo-European (indo1319), Atlantic-Congo (atla1278) and Afro-Asiatic (afro1255).

Table continues below


| abkh1242 | afro1255 | ainu1252 | araw1281 | atha1245 | atla1278 | aust1305 |
| --- | --- | --- | --- | --- | --- | --- |
| 1 | 13 | 1 | 1 | 2 | 19 | 3 |

Table continues below


| aust1307 | ayma1253 | basq1248 | chib1249 | drav1251 | eski1264 | hadz1240 |
| --- | --- | --- | --- | --- | --- | --- |
| 9 | 1 | 1 | 1 | 2 | 3 | 1 |

Table continues below


| indo1319 | japo1237 | jiva1245 | kore1284 | maya1287 | nilo1247 | nucl1710 |
| --- | --- | --- | --- | --- | --- | --- |
| 41 | 1 | 1 | 1 | 4 | 3 | 3 |

Table continues below


| otom1299 | pama1250 | pano1259 | sino1245 | taik1256 | ticu1244 | tupi1275 |
| --- | --- | --- | --- | --- | --- | --- |
| 1 | 1 | 1 | 9 | 1 | 1 | 1 |

| turk1311 | ural1272 | utoa1244 | yano1268 |
| --- | --- | --- | --- |
| 4 | 9 | 1 | 1 |

**Figure 41.** The distribution of languages across families. Only the 15 most represented families are explicitely shown, the others being gathered in the umbrella category ‘Other.’ Figure generated using `R` version 4.0.5 (2021-03-31) and package `ggplot2` (version 3.3.5).

**Figure 42.** Map of the main language families. Only the 15 most represented language families have individual colors. Figure generated using `R` version 4.0.5 (2021-03-31) and packages `ggplot2` (version 3.3.5), `maps` (version 3.3.0). Maps are using public domain data from the Natural Earth project as provided by the `R` package `maps`.

### 2.12.1 Putative origins of (macro-)families

The putative geographic origins of the language (macro-)families (latitude and longitude) were obtained from (Wichmann, Müller, & Velupillai, 2010), supplemented with information from the Glottolog (Hammarström, Bank, Forkel, & Haspelmath, 2018).

**Figure 43.** Origins of the putative origins of language (macro-)families. Figure generated using `R` version 4.0.5 (2021-03-31) and packages `ggplot2` (version 3.3.5), `maps` (version 3.3.0). Maps are using public domain data from the Natural Earth project as provided by the `R` package `maps`.

We also collected data corresponding to these locations for elevation, climate and ecology, humidity, distance to bodies of water and UV incidence; please note that (a) these locations are highly speculative, and (b) the information associated does not necessarily reflect the state of the world at the time when the proto-languages were spoken.

## 2.13 Macroarea

We collected the macroareas as given by the Glottolog (Hammarström, Bank, Forkel, & Haspelmath, 2018).

| Africa | Australia | Eurasia | North America | Papunesia | South America |
| --- | --- | --- | --- | --- | --- |
| 31 | 2 | 79 | 9 | 9 | 12 |

**Figure 44.** Map of populations in our sample highlighting their macroarea. Figure generated using `R` version 4.0.5 (2021-03-31) and packages `ggplot2` (version 3.3.5), `maps` (version 3.3.0). Maps are using public domain data from the Natural Earth project as provided by the `R` package `maps`.

## 2.14 Genetic distances between populations

Unfortunately, we could not obtain information about the *opsin* genes (*OPN1MW* and *OPN1LW*) for the populations in our sample. However, using a set of microhaplotypes and SNPs from the ALFRED database (Rajeevan, 2003), and the Arlequin (Excoffier & Lischer, 2010) software package, we computed the overall genetic distances between the populations. We imputed the missing values in the obtained distances matrix with the ultrametric methd (De Soete, 1984; Lapointe & Kirsch, 1995). Please see Appendix I. Distance matrices for more details.

Finally, we applied classic multi-dimensional scaling (`cmdscale`) to this imputed distance matrix, and we decided to retain the first 10 dimensions (resulting in a goodness-of-fit of 10.5%). These dimensions should represent an overall genetic similarity between our populations. For the statistical analyses, these were z-scored (thus, the relationship between raw and transformed values is linear).

## 2.15 Delaunay neighbors

We computed the Delaunay triangulation of our language locations taking into account the constraints to population movement imposed by large bodies of water; this is based on the method developed in Cysouw, Dediu, & Moran (2012), adapted by M. Tang and D. Dediu. This results in a list of neighbors for each population in our dataset.

**Figure 45.** Restricted Delaunay triangulation for the languages in our dataset (in a Pacific-centered view). The yellow dots are the language locations, the thin black lines the connections to their immediate neighbours, and the thick red lines represent barriers to movement. Figure generated using `R` version 4.0.5 (2021-03-31) and packages `ggplot2` (version 3.3.5), `maps` (version 3.3.0). Maps are using public domain data from the Natural Earth project as provided by the `R` package `maps`.

## 2.16 Input files and variables

These data can be found in the following input files (collected in the `./input_files` folder); “pre-processed” means produced by the associated `R` script `00_preprocess_data.R` (automatically, if needed); when transformed, the variable names usually takes the suffix "\_r“; when applied to families, the variable takes the suffix”\_family":

| Data | Input file | Pre-processed? | Variable name(s) | Transformed? | Also for families? |
| --- | --- | --- | --- | --- | --- |
| Populations and languages | `data_colors.csv` | No | *glottocode*, *L1* | No | No |
| Language family | `data_colors.csv` | No | *glottocode\_family*, *macro\_family* | No | Yes |
| Geographic locations | `data_colors.csv` | No | *latitude*, *longitude* | `cos()` | Yes |
| Elevation | `data_elevation.tsv` | From Mapzen | *elevation* | `log(1+)` | Yes |
| Climate and ecology | `data_climate.tsv` | From WorldClim | *clim\_PC1*, *clim\_PC2*, *clim\_PC3* | z-scored | Yes |
| Humidity | `data_chumidity.tsv` | From NOAA | *hum\_median*, *hum\_IQR* | No | Yes |
| Distance to bodies of water | `data_dist2water.tsv` | From OpenStreetMap | *dist2ocean*, *dist2lakes*, *dist2rivers*, *dist2water* | `log()` | Yes |
| UV incidence | `data_UV_incidence.tsv` | From NASA TOMS | *UV\_A\_mean*, *UV\_A\_sd*, *UV\_B\_mean*, *UV\_B\_sd*, *UV\_mean*, *UV\_sd* | z-scored | Yes |
| Log of population size | `data_colors.csv` | No | *log\_popSize* | No (already `log`’d) | No |
| Subsistence strategy | `data_colors.csv` | No | *subsistence* | No | No |
| Specific term for ‘blue’ | `data_colors.csv` | No | *exists\_blue* | No | No |
| Abnormal red/green color perception | `data_colors.csv` | No | *exists\_blue* | % → proportion (division by 100); 0% → 0.01% | No |
| Putative origins of (macro-)families | `data_colors.csv` | No | *latitude\_family*, *longitude\_family* | `cos()` | Yes |
| Macroarea | `data_colors.csv` | No | *macroarea* | No | Yes |
| Genetic distances | `data_cgenetics.tsv` | From ALFRED | *gen\_D1* .. *gen\_D10* | z-scored | No |
| Delaunay neighbors | computed | From languages’ geographic locations | *Delaunay\_N1*, *Delaunay\_N2*, .. | No | No |

# 3 Notes on methods

## 3.1 Bayesian mixed-effects regressions

For `brms`, to select the best model, we used Bayes factors, WAIC, LOO and KFOLD. Please note that, form `brms`, there might be differences between Bayes factors, on the one hand, and WAIC/LOO/KFOLD, on the other, due to the default use of improper priors (see, for example, https://stats.stackexchange.com/questions/407964/bayes-factors-and-predictive-accuracy-in-model-comparison-in-rstan-brms); therefore, we will both methods for model selection.

## 3.2 Mediation analysis

For the mediation analysis, we used a Bayesian approach as implemented by `brms`: in essence, given a treatment" \(T\) (for example, latitude), a mediator \(M\) (for example, UV incidence), as an outcome \(O\) (for example, the existence of a specific word for ‘blue’), the mediation model looks like:

**Figure 46.** Visual representation of a mediation, showing the individual estimates (edges, \(a\), \(b\) and \(c\)) and the effects (total, direct and indirect). We may use line style and color to differentiate ns, negative and positive edges. Likewise, we may report the total, direct and indirect effects on the diagram itself using colors to distinguish significant and ns effects. Figure generated using `R` version 4.0.5 (2021-03-31) and package `DiagrammeR` (version 1.0.6.1).

The *total effect* (i.e., the overall influence of \(T\) on \(O\), defined as \(a \cdot b + c\)) is decomposed into the *direct effect* (the arrow \(T \longrightarrow O\), defined as being equal to \(c\)) and the *indirect effect* “flowing” through \(M\) (\(T \longrightarrow M \longrightarrow O\), defined as \(a \cdot b\)). These are estimated by fitting the two mixed-effects regressions (with *family* and *macroarea* as random effects) to the data jointly:

\[
\begin{array}{l}
M \sim T + (1 | family) + (1 | macroarea) \\
O \sim T + M + (1 | family) + (1 | macroarea)
\end{array}
\]

Please note that in our plots, we may draw the edges differently depending on the “significance” and value of their estimates (\(a\), \(b\) and \(c\)).

# 4 Results

## 4.1 Spatial distribution of the data

How is our data distributed in geographical space? Is it random, or, if not, how does it deviate from spatial randomness? In this context, randomness means *complete spatial randomness* (CSR), where points are independent of each other, have the same likelihood of being found at any location, and their position is modeled by a Poisson distribution (Baddeley, Rubak, & Turner, 2015; Spielman, 2017).

Comparing the distribution of our data to a CSR process using the *χ*2 test based on quadrat counts, clearly rejects spatial randomness:

**Table 1.** Test of CSR using quadrat counts unsing *χ*2.


| Test statistic | df | P value | Alternative hypothesis |
| --- | --- | --- | --- |
| 401 | 74 | 4.202e-46 \* \* \* | two.sided |

**Table 2.** Test of CSR using quadrat counts unsing *χ*2 (conditional Monte Carlo).


| Test statistic | P value | Alternative hypothesis |
| --- | --- | --- |
| 401 | 0.001 \* \* \* | two.sided |

The same is found using the Kolmogorov-Smirnov test:

**Table 3.** Spatial Kolmogorov-Smirnov test of CSR in two dimensions.


| Test statistic | P value | Alternative hypothesis |
| --- | --- | --- |
| 0.5731 | 0 \* \* \* | two-sided |

Given that our locations are not randomly distributed, do they tend to be regularly spaced or clustered? The *G* function (using nearest neighbor distances) shows that the distances between the nearest neighbors are shorter than expected for a Poisson process, suggesting a clustering. The *F* function (using empty space distances) shows that the observed values are larger than the expected one, indicating that empty space distances in our empirical point pattern are shorter than for a Poisson process, suggesting again clustering. Finally, the *K* function (using pairwise distance) also suggests clustering.

**Figure 47.** The *G*, *F* and *K* functions (one per panel, from left to right). *r* is the distance between points. Expected distribution: blue; all others are the observed distributions with various edge correction methods (see help for `Gest` in package `spatstat`). Figure generated using `R` version 4.0.5 (2021-03-31) and package `spatstat` (version 2.2.0).

Thus, it is clear (and not surprising) that our datapoints are *significantly clustered*, reflecting, on the one hand, the actual patterning of linguistic diversity constrained by geography, ecology and climate, and, on the other, vagaries of data availability for the color vocabulary and the incidence of abnormal color perception.

## 4.2 Spatial autocorrelation

Spatial autocorrelation describes the degree to which locations are similar to each other at different distances, with respect to a given variable. We use here Moran’s *I* (Moran, 1950), which is a measure of global spatial autocorrelation, with either the inverse of the shortest geographical distance (“as the crow flies”) on the WGS84 ellipsoid, or the nearest neighbor distance on the Delaunay triangulation, as the weight matrix.

Using the inverse of the geographic distance, Moran’s *I* finds *significant positive autocorrelations* for the incidence of red/green abnormal color perception (observed = 0.1760 > expected = -0.0071, *p* = 0), the incidence of UV-A (observed = 0.4084 > expected = -0.0071, *p* = 0) and UV-B (observed = 0.4227 > expected = -0.0071, *p* = 0), the presence of a specific word for blue (observed = 0.2025 > expected = -0.0071, *p* = 0). Likewise, using the Delaunay neighbors, Moran’s *I* finds *significant positive autocorrelations* for the incidence of red/green abnormal color perception (observed = 0.4149 > expected = -0.0071, *p* = 4.123e-14), the incidence of UV-A (observed = 0.9092 > expected = -0.0071, *p* = 3.256e-59) and UV-B (observed = 0.9406 > expected = -0.0071, *p* = 4.748e-63), the presence of a specific word for blue (observed = 0.3051 > expected = -0.0071, *p* = 1.948e-08).

## 4.3 Hypothesis 1: UV → specific word for ‘blue’

Here we test the first hypothesis, linking UV incidence and the existence of a specific word for blue.

### 4.3.1 Variation between families and macroareas

First, we need to investigate the variation between families and macroareas, as we want to model them as random effects in our models.

The ICC of including separately family is 33.3%, and of macroarea is 25.3%; thus, we will include both in our models.

### 4.3.2 Potential predictors - blue

Here we look at the potential predictors of the existence of a specific word for ‘blue’ individually.

#### 4.3.2.1 UV → ‘blue’ (Brown & Lindsey, 2004; Lindsey & Brown, 2002)

Here we check if there is a relationship between measures of UV incidence and the existence of a word for ‘blue’; as per Lindsey & Brown (2002) and Brown & Lindsey (2004), this relationship should be *negative* (i.e., higher UV-B incidence should reduce the probability of a word for ‘blue’).

**Figure 48.** Probability of having a specific word for ‘blue’ function of UV incidence at the locations of the *languages*, showing the jittered data points, the densities (colored violins) and boxplots (black). Left: mean incidence across 1998, right: standard deviation of incidence over the days of 1998; top: UV-A, bottom: UV-B. Figure generated using `R` version 4.0.5 (2021-03-31) and package `ggplot2` (version 3.3.5).

Fitting individual Bayesian mixed-effects logistic regressions (with family and macroarea as random effects) predicting *exists\_blue* from the mean and standard deviation of the incidence of UV-A and UV-B for 1998 (both linear and quadratic effects), we found that:

- systematically, the quadratic effects do not contribute,
- the standard deviations (*UV\_A\_sd* and *UV\_B\_sd*) do not predict *exists\_blue* (*βUV\_A\_sd* = -0.38 (95%HDI = [-1.02, 0.29]), *p*(*β*=0) = 0.8; *βUV\_B\_sd* = -0.29 (95%HDI = [-0.87, 0.22]), *p*(*β*=0) = 0.85),
- the means (*UV\_A\_mean* and *UV\_B\_mean*) both have negative effects on *exists\_blue* (*βUV\_A\_mean* = -1.03 (95%HDI = [-1.65, -0.38]), *p*(*β*=0) = 0.0154, *p*(*β*<0) = 1; *βUV\_B\_mean* = -1.12 (95%HDI = [-1.75, -0.55]), *p*(*β*=0) = 0.0128, *p*(*β*<0) = 1),
- the two means are highly multicollinear (VIFUV\_A\_mean = 36.3, VIFUV\_B\_mean = 36.3),
- but *UV\_A\_mean* fits the data worse than *UV\_B\_mean* (Bayes factor = 0.31, LOO = -1.29 [SE=0.77], WAIC = -1.13 [SE=0.77], K-fold = -1.20 [SE=1.63]).

There is no (or very marginal) evidence that UV (A or B, mean or sd) incidence at the *origins of the language families* has any effect on the presence of a dedicated word for ‘blue.’

Therefore, we will only consider *UV\_B\_mean* (renamed to *UV-B* for shortness) in the following analyses.

#### 4.3.2.2 Latitude → ‘blue’

The effect of UV-B on the existence of a specific word for ‘blue’ (Brown & Lindsey, 2004; Lindsey & Brown, 2002) should be due to the physical negative relationship between the latitude of a location on the Earth’s surface and the mean annual UV-B radiation it receives; thus, it is justified to investigate the relationship between latitude and ‘blue.’ This relationship should specifically be *positive* (i.e., higher latitudes receive less UV-B incidence, which increase the chances of having a word for ‘blue’).

**Figure 49.** Probability of having a specific word for ‘blue’ function of latitude of the *languages*, showing the jittered data points, the densities (colored violins) and boxplots (black). From left to right: the linear effect of latitude and the quadratic effect of latitude (i.e., `latitude`2). Figure generated using `R` version 4.0.5 (2021-03-31) and package `ggplot2` (version 3.3.5).

When testing simultaneously the influence of *latitude* (linear effect) and *latitude*2 (quadratic effect) on the presence of a dedicated word for ‘blue,’ only the linear term has a positive effect: *βlatitude* = 4.39 (95%HDI = [0.82, 8.01]), posterior probability *p*(*β*=0) = 0.0677, *p*(*β*<0) = 0.994.

There is no evidence that the latitude of the *origins of language families* has any effect on the existence of a dedicated word for ‘blue.’

#### 4.3.2.3 Subsistence → ‘blue’

It has been suggested that various aspects of culture, including the subsistence mode, might have an effect on the existence of a dedicated word for ‘blue,’ and we should specifically expect that AGR populations have a *higher chance* of having a dedicated word for ‘blue.’

|  | has ‘blue’ | no ‘blue’ |
| --- | --- | --- |
| **HG** | 4 | 10 |
| **AGR** | 78 | 50 |

**Figure 50.** The relationship between subsistence type and the existence of a specific word for ‘blue.’ Figure generated using `R` version 4.0.5 (2021-03-31) and package `ggplot2` (version 3.3.5).

Subsistence does not seem to make a contribution to predicting the presence of a specific word for blue (*exists\_blue*): a Bayesian mixed-effects logistic regression of *exists\_blue* on *subsistence* [AGR vs HG] (with family and macroarea as random effects) has *βAGR-HG* = 0.97 (95%HDI = [-0.53, 2.68]), posterior probability *p*(*β*=0) = 0.6309, but this could be due to the very small proportion of HG populations in our sample (9.9%). Interestingly, the specific directional hypothesis of a positive effect of AGR is relatively well supported by the posterior probability *p*(*β*>0) = 0.8858.

#### 4.3.2.4 Elevation → ‘blue’

Arguably there may be a *positive* relationship between a location’s altitude above sea level and the average amount of UV-B it receives in a year.

**Figure 51.** Probability of having a specific word for ‘blue’ function of elevation (altitude) of the *languages*, showing the jittered data points, the densities (colored violins) and boxplots (black). From left to right: the linear effect of elevation and the quadratic effect of elevation (i.e., `elevation`2). Figure generated using `R` version 4.0.5 (2021-03-31) and package `ggplot2` (version 3.3.5).

However, there is no effect of *elevation* (linear effect) nor of *elevation*2 (quadratic effect), of the languages or of the origins of language families, on the presence of a dedicated word for ‘blue’ in our data, even when testing the specific directional hypothesis.

#### 4.3.2.5 Climate, ecology and humidity → ‘blue’

As opposed to the above, there do not seem to be good *a priori* reasons why climate, ecology and air humidity should influence the presence of a dedicated word for ‘blue.’

**Figure 52.** Probability of having a specific word for ‘blue’ function of climate, ecology and humidity at the location of the *languages*, showing the jittered data points, the densities (colored violins) and boxplots (black). From left to right and top to bottom: climate PC1, PC2 and PC3, median and IQR of humidity. Figure generated using `R` version 4.0.5 (2021-03-31) and package `ggplot2` (version 3.3.5).

However, when testing simultaneously the influence of the three climate PCs and of the median and IQR of air humidity on the presence of a dedicated word for ‘blue,’ only the first climate PC (*clim\_PC1*) has a positive effect: *βclim\_PC1* = 0.99 (95%HDI = [0.40, 1.57]), posterior probability *p*(*β*=0) = 0.03509.

There is no evidence that climate, ecology or humidity at the *origins of the language families* have any effect on the presence of a dedicated word for ‘blue.’

#### 4.3.2.6 Distance to water → ‘blue’

While we might imagine that closeness to large bodies of (clean and permanent) water might promote the use of a word for blue to describe their color (thus, a *negative* effect), this is, as far as we know, not been seriously suggested in the literature.

**Figure 53.** Probability of having a specific word for ‘blue’ function of distance to large bodies of water from the locations of the *languages*, showing the jittered data points, the densities (colored violins) and boxplots (black). From left to right and top to bottom, distances to: lakes, rivers, seas/oceans, and any type of body of water. Figure generated using `R` version 4.0.5 (2021-03-31) and package `ggplot2` (version 3.3.5).

However, when testing simultaneously the influence of the four distances to large bodies of water (both linear and squared) on the presence of a dedicated word for ‘blue,’ only the distance to lakes (*dist2lakes*) has a (linear) negative effect: *βdist2lakes* = -0.56 (95%HDI = [-0.87, -0.27]), posterior probability *p*(*β*=0) = 0.007372; the directional negative effect of distance is also very probable: *p*(*β*<0) = 1.

There is no evidence that distance to large bodies of water from the *origins of the language families* have any effect on the presence of a dedicated word for ‘blue.’

#### 4.3.2.7 (log) population size → ‘blue’

Again, there does not seem to be *a priori* suggestions for such a causal link, but we could imagine that larger populations, for various reasons, might have a higher chance of having a dedicated word for ‘blue.’

**Figure 54.** Probability of having a specific word for ‘blue’ function of (log) population size, showing the jittered data points, the densities (colored violins) and boxplots (black). Figure generated using `R` version 4.0.5 (2021-03-31) and package `ggplot2` (version 3.3.5).

However, it turns out that (log) population size has a strong positive effect on the presence of a dedicated word for ‘blue’: *βpopSize* = 0.31 (95%HDI = [0.16, 0.48]), posterior probability *p*(*β*=0) = 3.801e-07; the directional positive effect is also very probable: *p*(*β*>0) = 1.

#### 4.3.2.8 Genetic similarity → ‘blue’

While most cases of abnormal color perception do have a strong genetic basis, it is unclear if variation in the presence of a dedicated word for ‘blue’ between languages should correlate with the genetic similarity between the populations that speak those languages.

**Figure 55.** Probability of having a specific word for ‘blue’ function of between-populations genetic distances, showing the jittered data points, the densities (colored violins) and boxplots (black). From left to right and top to bottom, the first MDS components of the genetic distances matrix. Figure generated using `R` version 4.0.5 (2021-03-31) and package `ggplot2` (version 3.3.5).

Indeed, it turns out that several MDS dimensions of the genetic distances matrix influence the presence of a dedicated word for ‘blue’:

- *gen\_D1*: *βgen\_D1* = -0.88 (95%HDI = [-1.53, -0.31]), posterior probability *p*(*β*=0) = 0.1415;
- *gen\_D4*: *βgen\_D4* = -0.7 (95%HDI = [-1.34, -0.13]), posterior probability *p*(*β*=0) = 0.3919;
- *gen\_D6*: *βgen\_D6* = -0.59 (95%HDI = [-1.13, -0.02]), posterior probability *p*(*β*=0) = 0.505;
- *gen\_D7*: *βgen\_D7* = 0.87 (95%HDI = [0.35, 1.37]), posterior probability *p*(*β*=0) = 0.01421.

Geographically, these dimensions represent the following:

**Figure 56.** Map of the MDS dimensions of the genetic distances matrix that predict the existence of a specific word for ‘blue’ (color scale). Figure generated using `R` version 4.0.5 (2021-03-31) and package `ggplot2` (version 3.3.5).

- *gen\_D1* and *gen\_D4* seem to capture (to various degrees) low versus high latitudes,
- *gen\_D6* and *gen\_D4* seem to capture (to various degrees) Europe vs the rest of the world.

### 4.3.3 Mediation analyses

There are several possible mediated pathways connecting various variables and the existence of a dedicated word for ‘blue.’

#### 4.3.3.1 Latitude → UV-B → ‘blue’

**Figure 57.** Mediation analysis showing the total, direct and indirect effects, as well as the actual regression coefficients. Please note that because the outcome is binary, the direct and indirect effects may be on different scales. Figure generated using `R` version 4.0.5 (2021-03-31) and package `DiagrammeR` (version 1.0.6.1).

#### 4.3.3.2 Latitude → climate → ‘blue’

**Figure 58.** Mediation analysis showing the total, direct and indirect effects, as well as the actual regression coefficients. Please note that because the outcome is binary, the direct and indirect effects may be on different scales. Figure generated using `R` version 4.0.5 (2021-03-31) and package `DiagrammeR` (version 1.0.6.1).

#### 4.3.3.3 Latitude → subsistence → ‘blue’

**Figure 59.** Mediation analysis showing the total, direct and indirect effects, as well as the actual regression coefficients. Please note that because the outcome is binary, the direct and indirect effects may be on different scales. Figure generated using `R` version 4.0.5 (2021-03-31) and package `DiagrammeR` (version 1.0.6.1).

#### 4.3.3.4 Latitude → population size → ‘blue’

**Figure 60.** Mediation analysis showing the total, direct and indirect effects, as well as the actual regression coefficients. Please note that because the outcome is binary, the direct and indirect effects may be on different scales. Figure generated using `R` version 4.0.5 (2021-03-31) and package `DiagrammeR` (version 1.0.6.1).

#### 4.3.3.5 Climate → subsistence → ‘blue’

**Figure 61.** Mediation analysis showing the total, direct and indirect effects, as well as the actual regression coefficients. Please note that because the outcome is binary, the direct and indirect effects may be on different scales. Figure generated using `R` version 4.0.5 (2021-03-31) and package `DiagrammeR` (version 1.0.6.1).

#### 4.3.3.6 Climate → population size → ‘blue’

**Figure 62.** Mediation analysis showing the total, direct and indirect effects, as well as the actual regression coefficients. Please note that because the outcome is binary, the direct and indirect effects may be on different scales. Figure generated using `R` version 4.0.5 (2021-03-31) and package `DiagrammeR` (version 1.0.6.1).

#### 4.3.3.7 UV-B → subsistence → ‘blue’

**Figure 63.** Mediation analysis showing the total, direct and indirect effects, as well as the actual regression coefficients. Please note that because the outcome is binary, the direct and indirect effects may be on different scales. Figure generated using `R` version 4.0.5 (2021-03-31) and package `DiagrammeR` (version 1.0.6.1).

#### 4.3.3.8 UV-B → population size → ‘blue’

**Figure 64.** Mediation analysis showing the total, direct and indirect effects, as well as the actual regression coefficients. Please note that because the outcome is binary, the direct and indirect effects may be on different scales. Figure generated using `R` version 4.0.5 (2021-03-31) and package `DiagrammeR` (version 1.0.6.1).

#### 4.3.3.9 Subsistence → population size → ‘blue’

**Figure 65.** Mediation analysis showing the total, direct and indirect effects, as well as the actual regression coefficients. Please note that because the outcome is binary, the direct and indirect effects may be on different scales. Figure generated using `R` version 4.0.5 (2021-03-31) and package `DiagrammeR` (version 1.0.6.1).

#### 4.3.3.10 Climate → subsistence → population size

**Figure 66.** Mediation analysis showing the total, direct and indirect effects, as well as the actual regression coefficients. Please note that because the outcome is binary, the direct and indirect effects may be on different scales. Figure generated using `R` version 4.0.5 (2021-03-31) and package `DiagrammeR` (version 1.0.6.1).

#### 4.3.3.11 Extra: what’s going on with dist2lakes?

**Figure 67.** Mediation analysis showing the total, direct and indirect effects, as well as the actual regression coefficients. Please note that because the outcome is binary, the direct and indirect effects may be on different scales. Figure generated using `R` version 4.0.5 (2021-03-31) and package `DiagrammeR` (version 1.0.6.1).

**Figure 68.** Mediation analysis showing the total, direct and indirect effects, as well as the actual regression coefficients. Please note that because the outcome is binary, the direct and indirect effects may be on different scales. Figure generated using `R` version 4.0.5 (2021-03-31) and package `DiagrammeR` (version 1.0.6.1).

**Figure 69.** Mediation analysis showing the total, direct and indirect effects, as well as the actual regression coefficients. Please note that because the outcome is binary, the direct and indirect effects may be on different scales. Figure generated using `R` version 4.0.5 (2021-03-31) and package `DiagrammeR` (version 1.0.6.1).

#### 4.3.3.12 Interpretation

Thus, the data supports the following pathways:

**Figure 70.** The links supported by the mediation and regression analyses. Edges: solid blue = negative effects, solid red = positive effects, and dashed gray = null effects; the dashed red edge is borderline significant. Nodes: ‘blue’ in blue is the outcome, ‘UV-B’ in green is of particular interest here, light yellow have an effect on ‘blue,’ while light gray do not. This should *not* be interpreted as a path diagram! Figure generated using `R` version 4.0.5 (2021-03-31) and package `DiagrammeR` (version 1.0.6.1).

- *latitude* has no direct effect on *blue*, but its overall positive influence is mediated mostly by *UV-B* and, possibly, by *subsistence* and *dist2lakes*;
- similarly, *climate PC1* has no direct effect on *blue*, but its effect is mediated by *subsistence*;
- *subsistence* affects *blue* only through *population size*;
- *distance to lakes*, *UV-B* and *population size* have direct effects on *blue*.

### 4.3.4 Path analysis

We performed *path analysis* using `lavaan`, and we obtained the following model (built principally from *a priori* reasoning, but also incorporating some theoretically sound suggestions based on modification indices):

**Figure 71.** The path model with non-standardized coefficients, showing all estimated path estimates. Single-headed arrows represent regressions, double-headed arrows represent covariance and variance (when refering to the same node), and the arrows emerging from triangles are the intercepts; blue edges have negative estimates, red ones positive estimates. Figure generated using `R` version 4.0.5 (2021-03-31) and package `semPlot` (version 1.1.2).

**Figure 72.** The path model with standardized coefficients; conventions as above. Figure generated using `R` version 4.0.5 (2021-03-31) and package `semPlot` (version 1.1.2).

**Figure 73.** The path model with non-standardized coefficients, showing all path estimates with significance. Single-headed arrows represent regressions, while double-headed arrows represent covariance. Please note that this is not a “standard” SEM/path analysis diagram (see below for such a representation). Figure generated using `R` version 4.0.5 (2021-03-31) and package `lavaanPlot` (version 0.5.1).

**Figure 74.** The path model with standardized coefficients (see Grace & Bollen (2005) about pitfalls in interpreting such coefficients) showing only the significant (at the 0.05 level) path estimates with significance. Figure generated using `R` version 4.0.5 (2021-03-31) and package `lavaanPlot` (version 0.5.1).

This model fits the data well5: *χ*2(1) = 0.4, *p* = 0.528; CFI = 1.000, TLI = 1.045, NNFI = 1.045, RMSEA = 0.000 90%CI [0.000, 0.190].

We also created a path analysis model keeping only causal arrows where causality is fully assumed, either by our world’s knowledge (such as the impact of latitude on UV-B radiation) or by our hypothesis (see Potential predictors - blue); other more “uncertain” assumed causal arrows being replaced by correlation links.

**Figure 75.** The path model with standardized coefficients (see Grace & Bollen (2005) about pitfalls in interpreting such coefficients) showing only the significant (at the 0.05 level) path estimates with significance. Figure generated using `R` version 4.0.5 (2021-03-31) and package `lavaanPlot` (version 0.5.1).

This model also fits the data well: *χ*2(1) = 0.4, *p* = 0.528; CFI = 1.000, TLI = 1.045, NNFI = 1.045, RMSEA = 0.000 90%CI [0.000, 0.190].

Please note that:

- we coded the endogenous variables *blue* and *subsistence* as `ordered` (as “no” < “yes,” and ‘HG’ < ‘AGR,’ respectively) as per https://lavaan.ugent.be/tutorial/cat.html;
- by default, `lavaan` does not estimate the residual variances of categorical endogenous variables (here, *blue* and *subsistence*), and we did not change this behaviour.

With these, it can be seen that (the full model output is in Appendix II. Path analysis for ‘blue’):

- *latitude* seems to have a direct influence on all the other measures: not only on *UV-B* (-), but also on *climate PC1* (+), *distance to lakes* (-), and even *population size* (-), and (possibly) *subsistence* (-);
- *blue* is affected by *UV-B* (-), *distance to lakes* (-) and *population size* (+), the first strongly supporting the hypothesis of a physiological effect of UV-B incidence, and the latter supporting the suggestion that “cultural complexity” may favor more complex color vocabularies;
- *climate* influences only *population size* (+);
- *subsistence* influences *blue* only through *population size* (+).

(Please note thar including *longitude* in our model, so as to better capture the geographical patterning of linguistic diversity, did not seem to add anything.)

Thus, this path analysis model, despite its caveats (no modeling of measurement error, no modeling of the hierarchical structure due to language families and macroareas), does support the hypothesis of an important influence of UV-B incidence on the existence of a dedicated word for ‘blue’ above and beyond other factors (such as climate and subsistence strategy).

### 4.3.5 Predicting ‘blue’

We check how good are various techniques at predicting the existence of a dedicated word for ‘blue’ from a collection of potential predictors. On the one hand, we estimate this when using the full dataset (i.e., we use the full data both for fitting the model and for computing various measures of goodness of prediction), but also on how well these models generalize by repeatedly generating random training and testing subsets (containing 80% and 20% of the data, respectively; we stratify by macroarea and, except for Bayesian mixed-effects regressions, we do not use the language family; please note that random forests automatically sample the data).

#### 4.3.5.1 Multiple regression

We used Bayesian mixed-effects logistic regression to predict the existence of a specific word for ‘blue’ (*blue*) from all the potential predictors.

On the full dataset, Bayesian mixed effects logistic regression with family and macroarea as random effects and using all potential predictors as fixed effects, fits the data very well:

**Table 4.** Success (as %) on the full dataset using Bayesian mixed effects logistic regressions with all potential predictors.

| Predictors | Accuracy | Sensitivity | Specificity | Precision | Recall |
| --- | --- | --- | --- | --- | --- |
| All predictors | 89.4 | 91.4 | 86.9 | 90.2 | 91.4 |

After iterative manual simplification, we still fit the data very well:

**Table 5.** Success (as %) on the full dataset using Bayesian mixed effects logistic regressions with keeping only the predictors significantly contributing.

| Predictors | Accuracy | Sensitivity | Specificity | Precision | Recall |
| --- | --- | --- | --- | --- | --- |
| Set of predictors | 85.2 | 86.7 | 83.1 | 87.8 | 86.7 |

In this case, the retained predictors are:

**Table 6.** Retained predictors for Bayesian mixed effects logistic regressions following iterative maual simplification on the full dataset. The ROPE is [-0.18,0.18].

| Predictor | *β* (estimate) | *β* (95% HDI) | *p*ROPE |
| --- | --- | --- | --- |
| Intercept | -2.23 | [-5.11,0.74] | 0.031 |
| UVB\_r | -1.13 | [-1.91,-0.32] | 0 |
| log\_popSize | 0.37 | [0.19,0.56] | 0 |
| dist2lakes\_r | -0.55 | [-0.96,-0.17] | 0.005 |
| gen\_D7 | 0.70 | [0.23,1.14] | 0 |

When randomly splitting the dataset into 80% training/20% testing subsets 100 times, using all the potential predictors, we see that:

**Table 7.** Various measures of success at predicting *blue* using Bayesian mixed effects logistic regressions splitting the dataset randomly into 80% training/20% testing subsets, 100 times

| Predictors | Accuracy | Sensitivity | Specificity | Precision | Recall |
| --- | --- | --- | --- | --- | --- |
| All predictors | 73.1% ±7.9% | 77.6% ±9.9% | 67.9% ±12.1% | 75.7% ±10.1% | 77.6% ±9.9% |

**Figure 76.** Various measures of success using Bayesian mixed effects logistic regressions splitting the dataset randomly into 80% training/20% testing subsets, 100 times. Boxplots show the spread of the training/testing values, while the solid horizontal red lines show the values when using the full dataset. Figure generated using `R` version 4.0.5 (2021-03-31) and package `ggplot2` (version 3.3.5).

#### 4.3.5.2 Conditional inference trees

We used conditional inference trees (as implemented by `ctree()` in package `partykit`) to predict the existence of a specific word for ‘blue’ (*blue*) from all the potential predictors.

When using the full dataset, the decision trees explain it quite well:

**Figure 77.** Conditional inference trees for predicting *blue* using all the potential predictors. Please note that some predictors are transformed (the "\_r" suffix), the most important here being *macroarea*. Figure generated using `R` version 4.0.5 (2021-03-31) and package `partykit` (version 1.2.13).

**Table 8.** Success (as %) on the full dataset using conditional inference trees with all potential predictors.

| Predictors | Accuracy | Sensitivity | Specificity | Precision | Recall |
| --- | --- | --- | --- | --- | --- |
| All predictors | 84.5 | 87.5 | 80.6 | 85.4 | 87.5 |

It seems that the best predictor for having a word for ‘blue’ is the macroarea, with a distinction between Eurasian and Australian languages, on the one hand, and African, Papunesian and American languages, on the other, in both regions with small languages (≤ ≈1,000,000 speakers) having less ‘blue’; while in Eurasia and Australia UV-B incidence has a negative effect, in Africa, Papunesia and America, it is the distance to large bodies of water (lakes and oceans) that does.

When randomly splitting the dataset into 80% training/20% testing subsets 100 times, using all the potential predictors, we see that:

**Table 9.** Various measures of success at predicting *blue* using conditional inference trees splitting the dataset randomly into 80% training/20% testing subsets, 100 times.


| Predictors | Accuracy | Sensitivity | Specificity | Precision | Recall |
| --- | --- | --- | --- | --- | --- |
| All predictors | 69.6% ±8.4% | 73.8% ±10.5% | 65.0% ±13.9% | 74.4% ±12.1% | 73.8% ±10.5% |

**Figure 78.** Various measures of success using conditional inference trees splitting the dataset randomly into 80% training/20% testing subsets, 100 times. Boxplots show the spread of the training/testing values, while the solid horizontal red lines show the values when using the full dataset. Figure generated using `R` version 4.0.5 (2021-03-31) and package `ggplot2` (version 3.3.5).

#### 4.3.5.3 Random forests

We used random forests (as implemented by `randomForest()` in package `randomForest`) and conditional random forests (as implemented by `cforest()` in package `partykit`) to predict the existence of a specific word for ‘blue’ (*blue*) from all the potential predictors.

The (conditional) random forests are quite successful at predicting *blue*:

**Table 10.** Various measures of success at predicting *blue* using two random forest methods, 100 replications.


| Method | Accuracy | Sensitivity | Specificity | Precision | Recall |
| --- | --- | --- | --- | --- | --- |
| random forest | 75.9% ±1.1% | 79.8% ±1.2% | 70.9% ±1.3% | 78.0% ±1.4% | 79.8% ±1.2% |
| conditional random forest | 86.1% ±0.7% | 89.3% ±0.7% | 82.1% ±1.1% | 86.3% ±1.0% | 89.3% ±0.7% |

**Figure 79.** Various measures of success using (conditional) random forests with 100 replications. Figure generated using `R` version 4.0.5 (2021-03-31) and package `ggplot2` (version 3.3.5).

The importance of the predictors is:

**Figure 80.** Different measures of predictor importance using 100 replications. Left column: Accuracy-based predictor importance from random forests; this is a measure of the amount by which removing a variable decreases the accuracy thus higher values point to more relevant predictors. Column in the center: Gini-index-based predictor importance from random forests; this measures by how much the Gini impurity decrease when a variable is chosen to split a node (please note that only the relative values matter, and that there is a bias towards using numeric variables to split nodes). Right column: Unconditional predictor importance from conditional random forests; this is similar to the accuracy-based importance from random forests. Figure generated using `R` version 4.0.5 (2021-03-31) and package `ggplot2` (version 3.3.5).

It can be seen that:

1. *blue* is relatively well predicted, and that conditional random forests perform slightly better than “classic” random forests;
2. the various methods for estimating the predictor importance produce very similar results;
3. *population size* is the most important predictor
4. the importance of the other predictors is relatively stable, and suggests that *latitude*, *UV-B incidence*, *humidity*, *climate PC1* and *distance to lakes* tend to be the (next) most important.

#### 4.3.5.4 Support Vector Machines (SVM)

We used Support Vector Machines (SVMs, as implemented by `fit(...,model="svm")` in the `rminer` package) to predict the existence of a specific word for ‘blue’ (*blue*) from all the potential predictors. Here, we randomly split the dataset into a training (80% of the data points) and a testing (the remaining 20%) set (stratified on *blue*), repeated multiple times.

On the full data, SVMs are quite successful at predicting *blue*:

**Table 11.** Success (as %) on the full dataset using SVMs with all potential predictors.

| Predictors | accuracy | sensitivity | specificity | precision | recall |
| --- | --- | --- | --- | --- | --- |
| All predictors | 85.9 | 90.8 | 80.3 | 84.1 | 90.8 |

The predictors ordered by their importance (only those with importance > 0):

- with all predictors: *log\_popSize* (0.18), *clim\_PC3\_r* (0.15), *hum\_median* (0.15), *dist2ocean\_family\_r* (0.15), *clim\_PC1\_r* (0.09), *clim\_PC2\_r* (0.09), *dist2lakes\_r* (0.09), *dist2rivers\_r* (0.09)

When using 100 random training/testing subsets:

**Table 12.** Various measures of success at predicting *blue* using SVMs with 100 training/testing sets.


| Predictors | Accuracy | Sensitivity | Specificity | Precision | Recall |
| --- | --- | --- | --- | --- | --- |
| All predictors | 70.9% ±7.5% | 74.5% ±11.8% | 67.8% ±11.8% | 77.7% ±10.1% | 74.5% ±11.8% |

**Figure 81.** Various measures of success using SVMs with training/testing sets. Boxplots show the spread of the training/testing values, while the solid horizontal red lines show the values when using the full dataset. Figure generated using `R` version 4.0.5 (2021-03-31) and package `ggplot2` (version 3.3.5).

The importance of the predictors is:

**Figure 82.** Specificity-based predictor importance from SVMs using 100 training/testing sets. Figure generated using `R` version 4.0.5 (2021-03-31) and package `ggplot2` (version 3.3.5).

It can be seen that:

1. *blue* is relatively well predicted;
2. here too, *population size* is the most important predictor, but not exceedingly better than the next ones;
3. the next best predictors are *humidity*, *distance to oceans for the families*, *climate PC1*, *PC2* and *PC3*, *distance to lakes* and *UV-B incidence*.

#### 4.3.5.5 Interpretation

On the full dataset:

- all techniques predict *blue* very well from the available information, with very little difference between them
- consistently, the most relevant predictors are: *population size*, *UV-B*, *climate*/*humidity*, *latitude* and *distance to large bodies of standing water* (mostly lakes but also sometimes seas/oceans).

In what concerns generalizability (either when explicitly repeatedly splitting the data into training/testing subsets or internally using random forests):

- all techniques do pretty well and with comparable performance (as expected, lower than when using the full dataset for training and testing),
- the best are the conditional random forests and conditional trees, followed by Bayesian regression and SVMs.

Thus, the presence of a dedicated word for ‘blue’ is partly explained by population size and a few environmental variables concerning UV-B radiation, climate and distance to large bodies of standing water.

### 4.3.6 Conclusions: UV-B does influence the color vocabulary

Using various types of methods, we do find, as predicted by Lindsey & Brown (2002) and Brown & Lindsey (2004) that the presence of a dedicated word for ‘blue’ is influenced negatively by the amount of UV-B incident radiation (i.e., that higher levels of UV-B radiation reduce the probability that a language has such a word). Interestingly, different techniques vary in their support for an influence of measures of “cultural complexity” (such as subsistence strategy), but there is at least a hint of a positive relationship. The mediation analysis and path analysis suggest that the effect of subsistence is mediated by population size. Likewise, climate, ecology and humidity might affect the presence of a dedicated word for ‘blue,’ with drier, higher seasonality increasing the probability of ‘blue.’ Finally, there is an a priori surprising negative effect of distance to large bodies of standing water (lakes, in particular).

## 4.4 Hypothesis 2: UV incidence and abnormal color perception

Here we test the second hypothesis (Brown & Lindsey, 2004), linking UV incidence and the population frequency of abnormal color perception:

**Figure 83.** Graphical representation of Hypothesis 2. Figure generated using `R` version 4.0.5 (2021-03-31) and package `DiagrammeR` (version 1.0.6.1).

With our variables (and strictly following the hypothesis), this becomes:

**Figure 84.** Graphical representation of Hypothesis 2 with our measures as a SEM diagram. Figure generated using `R` version 4.0.5 (2021-03-31) and package `DiagrammeR` (version 1.0.6.1).

However, we do not have enough data to model the two latent variables, so our graph reduces to the following path analysis:

**Figure 85.** Graphical representation of Hypothesis 2 with our measures as a path analysis diagram. Figure generated using `R` version 4.0.5 (2021-03-31) and package `DiagrammeR` (version 1.0.6.1).

### 4.4.1 Variation between families and macroareas

First, we need to investigate the variation between families and macroareas, as we want to model them as random effects in our models.

The ICC of including separately family is 21.7%, and of macroarea is 21.2%; thus, we will include both in our models.

### 4.4.2 Potential predictors

Here we look at the potential predictors individually.

#### 4.4.2.1 ‘blue’ → daltonism

**Figure 86.** Population frequency of daltonism (in %) function of having a specific word for ‘blue,’ showing the jittered data points, the densities (colored violins) and boxplots (black). Figure generated using `R` version 4.0.5 (2021-03-31) and package `ggplot2` (version 3.3.5).

The existence of a dedicated word for ‘blue’ (*exists\_blue*) has a significant positive influence on *daltonism*: *βblue:yes-no* = 0.61 (95%HDI = [0.33, 0.88]), *p*(*β*=0) = 0.0004, *p*(*β*>0) = 1); and fits the data much better than the null model (Bayes factor = 0.0027, LOO = -7.45 [SE=5.77], WAIC = -7.97 [SE=5.84], K-fold = -4.63 [SE=5.41]).

#### 4.4.2.2 daltonism → ‘blue’

Conversely, *daltonism* has a positive effect on the existence of a dedicated word for ‘blue’ (*exists\_blue*): *βdaltonism* = 16.57 (95%HDI = [-2.93, 41.15]), *p*(*β*=0) = 0.17, *p*(*β*>0) = 0.95); and fits the data better than the null model (Bayes factor = 0.23, LOO = -2.70 [SE=2.09], WAIC = -2.59 [SE=2.09], K-fold = -6.12 [SE=2.38]).

#### 4.4.2.3 UV-B → daltonism

**Figure 87.** Population frequency of daltonism (in %) function of UV-B incidence, showing the data points and the linear and LOESS trends (with 95% CIs). Figure generated using `R` version 4.0.5 (2021-03-31) and package `ggplot2` (version 3.3.5).

UV-B incidence has a significant negative linear effect on *daltonism* (the quadratic effect is clearly not important): *βUVB* = -0.3 (95%HDI = [-0.45, -0.15]), *p*(*β*=0) = 0.083, *p*(*β*<0) = 1); and fits the data much better than the null model (Bayes factor = 0.03, LOO = -5.62 [SE=3.70], WAIC = -6.19 [SE=3.69], K-fold = 0.00 [SE=0.00]).

#### 4.4.2.4 Latitude → daltonism

**Figure 88.** Population frequency of daltonism (in %) function of UV-B incidence, showing the data points and the linear and LOESS trends (with 95% CIs). Figure generated using `R` version 4.0.5 (2021-03-31) and package `ggplot2` (version 3.3.5).

*Latitude* has a significant positive linear effect on *daltonism* (the quadratic effect is clearly not important): *βlatitude* = 1.69 (95%HDI = [0.78, 2.64]), *p*(*β*=0) = 0.0076, *p*(*β*<0) = 0); and fits the data much better than the null model (Bayes factor = 0.0093, LOO = -5.20 [SE=3.09], WAIC = -5.88 [SE=3.18], K-fold = -4.26 [SE=4.61]).

#### 4.4.2.5 daltonism and genetic distances

**Figure 89.** Population frequency of daltonism (in %) function of genetic distance between populations. Figure generated using `R` version 4.0.5 (2021-03-31) and package `ggplot2` (version 3.3.5).

Interestingly, the population frequency of daltonism is only weakly correlated with the inter-population genetic distances: a Bayesian mixed-effects quadratic Beta regression of daltonism with family and macroarea as random effects and the first 10 MDS dimensions of the ultrametrically-imputed genetic distances matrix with manual simplification is better than the null model (Bayes factor = 5.1e+03, LOO = -8.44 [SE=4.93], WAIC = -9.44 [SE=5.07], K-fold = -8.08 [SE=4.74]), has R2= 46.3% ±4.7%, and the retained predictors are:

**Table 13.** Retained predictors ; the ROPE is [-0.1,0.1].

| Predictor | *β* (estimate) | *β* (95% HDI) | *p*ROPE |
| --- | --- | --- | --- |
| Intercept | -3.82 | [-4.56,-3.05] | 0 |
| gen\_D1 | -0.12 | [-0.19,-0.05] | 0.26 |
| gen\_D8 | -0.08 | [-0.17,-0.01] | 0.65 |
| Igen\_D2E2 | -0.06 | [-0.1,-0.02] | 1 |
| Igen\_D5E2 | 0.06 | [0,0.12] | 0.91 |

Likewise, the Mantel correlations between the Euclidean distances between population frequencies in daltonism and ultrametrically-imputed genetic distances matrix are weak but significant (using 1000 permutations): with Pearson *r*=0.078, *p*=0.002, and with Spearman *ρ*=0.077, *p*=0.001.

### 4.4.3 Mediation analyses

#### 4.4.3.1 UV-B → ‘blue’ → daltonism

**Figure 90.** Mediation analysis showing the total, direct and indirect effects, as well as the actual regression coefficients. Please note that because the mediator is binary, the direct and indirect effects may be on different scales. Figure generated using `R` version 4.0.5 (2021-03-31) and package `DiagrammeR` (version 1.0.6.1).

Thus, as suggested, *daltonism* is negatively affected by *UV-B* incidence (total effect) both directly and indirectly, mediated by *blue*; in particular, the frequency of daltonism is higher is populations whose language has a dedicated word for ‘blue.’

#### 4.4.3.2 latitude → UV-B → daltonism

**Figure 91.** Mediation analysis showing the total, direct and indirect effects, as well as the actual regression coefficients. Please note that because the mediator is binary, the direct and indirect effects may be on different scales. Figure generated using `R` version 4.0.5 (2021-03-31) and package `DiagrammeR` (version 1.0.6.1).

#### 4.4.3.3 latitude → ‘blue’ → daltonism

**Figure 92.** Mediation analysis showing the total, direct and indirect effects, as well as the actual regression coefficients. Please note that because the mediator is binary, the direct and indirect effects may be on different scales. Figure generated using `R` version 4.0.5 (2021-03-31) and package `DiagrammeR` (version 1.0.6.1).

#### 4.4.3.4 Interpretation

**Figure 93.** Graphical representation of the links supported by the mediation and regression analyses. Edges: solid blue = negative effects, solid red = positive effects, and dashed gray = non-significant effects; the dashed red edge is borderline significant. Nodes: ‘blue’ in blue is the outcome, ‘UV-B’ in green is of particular interest here. This should *not* be interpreted as a path diagram! Figure generated using `R` version 4.0.5 (2021-03-31) and package `DiagrammeR` (version 1.0.6.1).

The mediation analysis for the hypothesis 1 also showed that distance to lakes, population size, climate and subsistence could potentially be included in this diagram for their effects on blue. However, as they do not affect *daltonism* here, we did not include them in the diagram.

### 4.4.4 Path analysis

Here we fit a path analysis model that is strictly following hypothesis 2 (i.e., we exclude other potential factors):

**Figure 94.** The path model with non-standardized coefficients, showing all estimated path estimates. Single-headed arrows represent regressions, double-headed arrows represent covariance and variance (when refering to the same node), and the arrows emerging from triangles are the intercepts; blue edges have negative estimates, red ones positive estimates. Figure generated using `R` version 4.0.5 (2021-03-31) and package `semPlot` (version 1.1.2).

**Figure 95.** The path model with standardized coefficients; conventions as above. Figure generated using `R` version 4.0.5 (2021-03-31) and package `semPlot` (version 1.1.2).

**Figure 96.** The path model with non-standardized coefficients, showing all path estimates with significance. Single-headed arrows represent regressions, while double-headed arrows represent covariance. Please note that this is not a “standard” SEM/path analysis diagram (see below for such a representation). Figure generated using `R` version 4.0.5 (2021-03-31) and package `lavaanPlot` (version 0.5.1).

**Figure 97.** The path model with standardized coefficients (see Grace & Bollen (2005) about pitfalls in interpreting such coefficients) showing only the significant (at the 0.05 level) path estimates with significance. Figure generated using `R` version 4.0.5 (2021-03-31) and package `lavaanPlot` (version 0.5.1).

This model fits the data well: *χ*2(1) = 0.1, *p* = 0.804; CFI = 1.000, TLI = 1.069, NNFI = 1.069, RMSEA = 0.000 90%CI [0.000, 0.141].

As *subsistence* could potentially be linked to *colour blindness* rate, as observed by (Salzano (1964); Salzano (1961); Richard H. Post (1963); R. H. Post (1962) Pickford (1963) ; H. Kalmus, Degaray, Rodarte, & Cobo (1964); H. Kalmus, Amir, Levine, Barak, & Goldschmidt (1961); H. Kalmus (1957); Junqueira, Kalmus, & Wishart (1957) ; Garth (1933); Adam (1969)), we also created a path analysis model including subsistence:

**Figure 98.** The path model with standardized coefficients (see Grace & Bollen (2005) about pitfalls in interpreting such coefficients) showing only the significant (at the 0.05 level) path estimates with significance. Figure generated using `R` version 4.0.5 (2021-03-31) and package `lavaanPlot` (version 0.5.1).

This model also fits the data well: *χ*2(1) = 1.2, *p* = 0.28; CFI = 0.998, TLI = 0.990, NNFI = 0.990, RMSEA = 0.034 90%CI [0.000, 0.229].

The full model outputs are in Appendix III. Path analysis for “daltonism”), but we can see that:

- as above, *latitude* affects *UV-B* (-) and *blue* (-),
- and *daltonism* is affected by:
  - in the absence of *subsistence*, by *blue* (+) and by *UV-B* (-),
  - but, when *subsistence* is added, by *subsistence* (+) and *UV-B* (-).

### 4.4.5 Predicting daltonism

We check how good are various techniques at predicting the population frequency of daltonism from a collection of potential predictors. As for ‘blue,’ we estimate this on the full dataset and on random training and testing subsets.

#### 4.4.5.1 Multiple regression

On the full dataset, Bayesian mixed effects logistic regression with family and macroarea as random effects and using all potential predictors as fixed effects, fits the data very well: R2 = 52.9%, RMSE = 0.033. After iterative manual simplification, we still fit the data very well (R2 = 52.8%, RMSE = 0.032), and the retained predictors are:

**Table 14.** Retained predictors for Bayesian mixed effects logistic regressions following iterative maual simplification on the full dataset. The ROPE is [-0.1,0.1].

| Predictor | *β* (estimate) | *β* (95% HDI) | *p*ROPE |
| --- | --- | --- | --- |
| Intercept | -3.59 | [-4.26,-2.98] | 0 |
| longitude\_r | 0.34 | [0.08,0.62] | 0.014 |
| clim\_PC1\_r | 0.17 | [0,0.31] | 0.19 |
| dist2water\_r | -0.10 | [-0.17,-0.02] | 0.54 |
| exists\_blueyes | 0.36 | [0.1,0.67] | 0.0024 |
| gen\_D8\_r | -0.15 | [-0.26,-0.04] | 0.15 |

When randomly splitting the dataset into 80% training/20% testing subsets 100 times, using all the potential predictors, we obtain R2 = 47.5% ±5.5%, and RMSE = 0.040 ±0.004.

**Figure 99.** Various measures of success using Bayesian mixed effects logistic regressions splitting the dataset randomly into 80% training/20% testing subsets, 100 times. Boxplots show the spread of the training/testing values, while the solid horizontal lines show the values when using the full dataset. Figure generated using `R` version 4.0.5 (2021-03-31) and package `ggplot2` (version 3.3.5).

#### 4.4.5.2 Conditional inference trees

We used conditional inference trees (as implemented by `ctree()` in package `partykit`) to predict the population frequency of *daltonism* from all the potential predictors:

On the full dataset, the conditional inference tree using all potential predictors as fixed effects, fits the data very well: R2 = 54.3%, RMSE = 0.017, and the tree is:

**Figure 100.** Conditional inference trees for predicting *daltonism* using all the potential predictors. Figure generated using `R` version 4.0.5 (2021-03-31) and package `partykit` (version 1.2.13).

When randomly splitting the dataset into 80% training/20% testing subsets 100 times, using all the potential predictors, we obtain R2 = 14.8% ±22.9%, and RMSE = 0.023 ±0.003.

**Figure 101.** Various measures of success using Bayesian mixed effects logistic regressions splitting the dataset randomly into 80% training/20% testing subsets, 100 times. Boxplots show the spread of the training/testing values, while the solid horizontal lines show the values when using the full dataset. Figure generated using `R` version 4.0.5 (2021-03-31) and package `ggplot2` (version 3.3.5).

#### 4.4.5.3 Random forests

We used random forests (as implemented by `randomForest()` in package `randomForest`) and conditional random forests (as implemented by `cforest()` in package `partykit`) to predict the existence of a specific word for ‘blue’ (*blue*) from all the potential predictors:

On the full dataset, the (conditional) random forests using all potential predictors as fixed effects, fit the data very well:

- random forests: R2 = 33.3% ±0.8%, RMSE = 0.021 ±0.000
- conditional random forests: R2 = 54.2% ±0.2%, RMSE = 0.017 ±0.000.

**Figure 102.** Various measures of success using random forests (RF) and conditional random forests (CRF). Figure generated using `R` version 4.0.5 (2021-03-31) and package `ggplot2` (version 3.3.5).

The importance of the predictors is:

**Figure 103.** Predictor importance from random forests using 100 replications using random forests (left: accuracy-based, middle: Gini-index based) and conditional random forests (right). Figure generated using `R` version 4.0.5 (2021-03-31) and package `ggplot2` (version 3.3.5).

#### 4.4.5.4 SVM

We used Support Vector Machines (SVMs, as implemented by `fit(...,model="svm")` in the `rminer` package) to predict the population frequency of daltonism (*daltonism*) from all the potential predictors.

On the full dataset, SVMs using all potential predictors, fits the data very well: R2 = 68.5%, RMSE = 0.014, and the variable importance is (only those with importance > 0):

*gen\_D3\_r* (0.116), *latitude\_r* (0.106), *log\_popSize* (0.093), *dist2lakes\_r* (0.088), *clim\_PC1\_r* (0.084), *dist2ocean\_r* (0.056), *longitude\_r* (0.048), *UVB\_r* (0.045), *exists\_blue* (0.045), *gen\_D4\_r* (0.042), *dist2rivers\_r* (0.04), *gen\_D8\_r* (0.036), *gen\_D1\_r* (0.036), *gen\_D7\_r* (0.033), *elevation\_r* (0.021), *hum\_median* (0.019), *gen\_D5\_r* (0.019), *subsistence* (0.014), *gen\_D10\_r* (0.013), *dist2water\_r* (0.009), *hum\_IQR* (0.006), *gen\_D9\_r* (0.006), *clim\_PC2\_r* (0.006), *gen\_D2\_r* (0.005), *macroarea* (0.005), *gen\_D6\_r* (0.005), *clim\_PC3\_r* (0.003)

When randomly splitting the dataset into 80% training/20% testing subsets 100 times, using all the potential predictors, we obtain R2 = 31.9% ±18.7%, and RMSE = 0.020 ±0.003.

**Figure 104.** Various measures of success using SVMs splitting the dataset randomly into 80% training/20% testing subsets, 100 times. Boxplots show the spread of the training/testing values, while the solid horizontal lines show the values when using the full dataset. Figure generated using `R` version 4.0.5 (2021-03-31) and package `ggplot2` (version 3.3.5).

The importance of the predictors is:

**Figure 105.** Specificity-based predictor importance from SVMs using 100 training/testing sets. Figure generated using `R` version 4.0.5 (2021-03-31) and package `ggplot2` (version 3.3.5).

### 4.4.6 Conclusions: daltonism, UV-B and the color vocabulary

When focusing on the evolutionary hypothesis (Brown & Lindsey, 2004) linking UV-B incidence and the population frequency of red/green abnormal color perception (“daltonism”), we found that, indeed, this is supported by our data. The overall negative impact of UV-B is largely mediated by the existence of a dedicated word for ‘blue,’ supporting the conjecture that already experiencing UV-B-induced blue/green perception loss generates selective pressures against red/green abnormal color perception. Interestingly, the overall genetic distance between populations is not a good predictor of the frequency of daltonism, again suggesting that daltonism is affected by other evolutionary forces than just drift and demographic history.

# 5 Overall conclusions and discussion

Overall, our extended dataset and more advanced methods do provide support for both hypotheses: on the one hand, the presence of a dedicated word for ‘blue’ is influenced negatively by the amount of UV-B incident radiation (hypothesis 1), and, on the other, that there is a negative effect of UV-B on the population frequency of red/green abnormal color perception (“daltonism”) that is largely mediated by the existence of a dedicated word for ‘blue’ (hypothesis 2). Moreover, our analyses suggest new findings: various proxies of “cultural complexity” (population size, subsistence strategy) seem to also have an effect on the presence of a word for blue, supporting the idea that more “complex” cultures favor more “complex” color vocabularies. Surprisingly, we also found that drier climates with higher seasonality may increase the probability of a dedicated word for ‘blue,’ as does spatial closeness to large bodies of standing water (in particular, lakes), suggesting an effect of the environment on the color vocabulary probably mediated by the salience and importance of “water” and the open sky. We also found that the overall genetic distance between populations is not a good predictor of the frequency of daltonism, suggesting that variation in daltonism between groups is not fully explained by genetic drift and demographic history, but may be under selective pressures.

Thus, we found that the color vocabulary is shaped by environmental factors acting on the individual speakers, generating biases that are amplified by the repeated use and transmission of language in communities of similarly affected individuals. This is akin to other such cases of bias amplification to the level of cross-linguistic diversity (Dediu, Janssen, & Moisik, 2017), either (presumably) rooted in genetics (Dediu & Ladd, 2007; Dediu & Moisik, 2019; Moisik & Dediu, 2017) or emerging during the lifetime of the individuals due to environmental or cultural factors (Blasi et al., 2019). But what sets this case apart, is that the cross-linguistic effects of UV-B incidence on the color vocabulary (presumably mediated by physiologic effects accrued during the life time of the speakers) are one manifestation of group-wide acquired color (green/blue) abnormal perception, the other being environmentally-mediated differential evolutionary pressures between groups against the incidence of congenial inherited abnormal color (red/green) perception.

# 6 Acknowledgements

Thanks to the Huma-Num for access to their computer cluster during the development of this analysis script. DD was funded by an IDEXLYON (16-IDEX-0005) Fellowship grant (2018-2021), MJ by the NSCO doctoral school of Lyon. The project was indirectly supported by the LabEx ASLAN (ANR-10-LABX-0081) of the University of Lyon within the program Investissements d’Avenir (ANR-11-IDEX-0007) of the French National Research Agency (ANR).

# 7 Author contributions

AM and DD designed research. EM extended the database and performed initial analysis. DD and MJ further extended the database and performed analyses. DD, MJ and AM wrote first draft of the paper. All authors contributed to the paper, read and approved it.

# 8 Appendices

## 8.1 Appendix I. Distance matrices

We use the *fixation Index* (FST) as a measure of the genetic distance between pairs of populations, with high values indicating a high differentiation betwen populations; there are multiple approaches (see, for example, Wikipedia, Holsinger & Weir (2009)), but we used here the method implemented in `Arlequin 3.5` (Excoffier & Lischer, 2010) as detailed below.

As *primary data*, we used a subset of the ALFRED (The ALlele FREquency Database) (Rajeevan, 2003), the FROG-kb (Forensic Resource/Reference on Genetics knowledge base), aimed at forensic applications. More precisely, we downloaded (as of spring 2020) the following files:

| Panel | File (hyperlink) | N | #SNPs6 | #MHs7 |
| --- | --- | --- | --- | --- |
| Microhaplotypes | *Microhap\_alleleF\_198.txt* | 96 | 0 | 198 |
| Seldin’s list of 128 AISNPs | *Seldin128\_alleleF.txt* | 70 | 128 | 0 |
| SNPforID 34-plex | *SNPForId34\_alleleF.txt* | 53 | 34 | 0 |
| KiddLab - Set of 55 AISNPs | *KiddLab55\_alleleF.txt* | 139 | 55 | 0 |
| Kayser’s set of 24 Ancestry Informative Markers | *Kayser24\_alleleF.txt* | 73 | 24 | 0 |
| Daniele Podini’s list of 32 AISNPs | *Podini32\_alleleF.txt* | 111 | 29 | 0 |
| Eurasiaplex 23 SNP Panel | *Eurasiaplex23\_alleleF.txt* | 76 | 23 | 0 |
| Nievergelt’s Set of 41AIMs | *Nievergelt41\_alleleF.txt* | 123 | 41 | 0 |
| Overlap set of AISNPs | *Overlap44\_alleleF.txt* | 72 | 44 | 0 |
| Li’s panel of 74 AIMS | *Li74\_alleleF.txt* | 67 | 73 | 0 |

These text files were combined into a single file, and we used a custom `R` script (available online at `mathjoss/Alfred2FST/alfredtxt2arlequin.R`) to extract one `Arlequin file` (`.arp`) per microhaplotype and per SNP. These `.arp` files were then processed with `Arlequin 3.5` (Excoffier & Lischer, 2010) (the parameters can be found in mathjoss/Alfred2FST/arl\_run.ars), using the output tables `Population average pairwise difference` (and, more precisely, the *corrected average pairwise difference* component), one per input `.arp` file. We averaged these tables using two other custom `R` scripts (available at mathjoss/Alfred2FST/mean\_all\_files.R and mathjoss/Alfred2FST/mean\_all\_files\_part2.R). This procedure resulted in average pairwise distance between 145 unique populations, using 96 unique microhaplotypes and 382 unique SNP.

As there is missing data, we used the *additive* and the *ultrametric* methods of data imputation for distance matrices (De Soete, 1984; Lapointe & Kirsch, 1995), followed by *Multi-Dimensional Scaling* (*MDS*); see the `R` script at mathjoss/Alfred2FST/extract\_MDS.R for details.

Finally, we mapped manually the 145 unique populations with FST distances to the 142 populations in our primary dataset `data_colors.csv`, but the mapping is far from trivial and unambiguous: for example, some ALFRED populations are rather large and imprecise (e.g., “African Americans,” “Indian Mixed” …). Consequently, we applied the following procedure:

1. Exact population were linked to each other (e.g., “Basque” and “Yoruba” are present in both);
2. For populations in the primary dataset but not present in the FST distances matrix, we selected its most appropriate general category (e.g. for “Czech” we used “Europeans Mixed,” and for “Gujarati,” “Indian mixed”);
3. If there is no appropriate general category, we attempted to find the closest matching population in the FST distances matrix (generally based on geographic distance and demographic history, as inferred from various sources).

The results of the first two matching rules (1 & 2) are in the *genetic\_incomplete* column of the `data_colors.csv` primary dataset, while those of all the three rules (1, 2 & 3) are in the *genetic\_full* column – we used this “fully” matched column for further analyses.

## 8.2 Appendix II. Path analysis for ‘blue’

The full output of this model, with all the fit indices, is:

```
lavaan 0.6-9 ended normally after 119 iterations

  Estimator                                       DWLS
  Optimization method                           NLMINB
  Number of model parameters                        30
                                                      
  Number of observations                           142
                                                      
Model Test User Model:
                                              Standard      Robust
  Test Statistic                                 0.398       0.398
  Degrees of freedom                                 1           1
  P-value (Chi-square)                           0.528       0.528
  Scaling correction factor                                  1.000
  Shift parameter                                            0.000
       simple second-order correction                             

Parameter Estimates:

  Standard errors                           Robust.sem
  Information                                 Expected
  Information saturated (h1) model        Unstructured

Regressions:
                   Estimate  Std.Err  z-value  P(>|z|)
  blue ~                                              
    uvb     (b_bu)   -0.780    0.339   -2.301    0.021
    d2l     (b_bd)   -0.133    0.051   -2.632    0.008
    subs    (b_bs)    0.190    0.119    1.603    0.109
    clim    (b_bc)   -0.021    0.208   -0.099    0.921
    lat     (b_bl)   -1.436    2.049   -0.701    0.483
    psiz    (b_bp)    0.094    0.022    4.209    0.000
  uvb ~                                               
    lat     (b_ul)   -5.671    0.151  -37.552    0.000
  clim ~                                              
    lat     (b_cl)    5.100    0.298   17.113    0.000
  d2l ~                                               
    lat     (b_dl)   -4.088    0.781   -5.233    0.000
  subs ~                                              
    lat     (b_sl)   -6.353    2.509   -2.532    0.011
    d2l     (b_sd)   -0.127    0.070   -1.819    0.069
    uvb     (b_su)   -0.906    0.394   -2.301    0.021
    clim    (b_sc)   -0.085    0.262   -0.323    0.746
  psiz ~                                              
    subs    (b_ps)    1.760    0.448    3.927    0.000
    lat     (b_pl)  -11.279    5.532   -2.039    0.041
    d2l     (b_pd)   -0.397    0.195   -2.040    0.041
    uvb     (b_pu)   -0.204    0.914   -0.223    0.823
    clim    (b_pc)    2.350    0.594    3.958    0.000

Covariances:
                   Estimate  Std.Err  z-value  P(>|z|)
 .uvb ~~                                              
   .clim             -0.033    0.014   -2.447    0.014
   .d2l               0.070    0.038    1.840    0.066

Intercepts:
                   Estimate  Std.Err  z-value  P(>|z|)
   .blue              0.000                           
   .uvb               0.927    0.047   19.793    0.000
   .clim             -0.834    0.056  -14.841    0.000
   .d2l               4.431    0.177   24.966    0.000
   .subs              0.000                           
   .psiz             19.786    1.297   15.251    0.000

Thresholds:
                   Estimate  Std.Err  z-value  P(>|z|)
    blue|t1           0.037    0.617    0.060    0.952
    subs|t1          -2.829    0.554   -5.102    0.000

Variances:
                   Estimate  Std.Err  z-value  P(>|z|)
   .blue              0.629                           
   .uvb               0.083    0.013    6.239    0.000
   .clim              0.257    0.029    8.756    0.000
   .d2l               2.542    0.333    7.625    0.000
   .subs              0.878                           
   .psiz             10.536    1.154    9.129    0.000

Scales y*:
                   Estimate  Std.Err  z-value  P(>|z|)
    blue              1.000                           
    subs              1.000
```

```
                         npar                          fmin 
                       30.000                         0.001 
                        chisq                            df 
                        0.398                         1.000 
                       pvalue                  chisq.scaled 
                        0.528                         0.398 
                    df.scaled                 pvalue.scaled 
                        1.000                         0.528 
         chisq.scaling.factor                baseline.chisq 
                        1.000                       215.483 
                  baseline.df               baseline.pvalue 
                       15.000                         0.000 
        baseline.chisq.scaled            baseline.df.scaled 
                      169.499                        15.000 
       baseline.pvalue.scaled baseline.chisq.scaling.factor 
                        0.000                         1.298 
                          cfi                           tli 
                        1.000                         1.045 
                         nnfi                           rfi 
                        1.045                         0.972 
                          nfi                          pnfi 
                        0.998                         0.067 
                          ifi                           rni 
                        1.003                         1.003 
                   cfi.scaled                    tli.scaled 
                        1.000                         1.058 
                   cfi.robust                    tli.robust 
                           NA                            NA 
                  nnfi.scaled                   nnfi.robust 
                        1.058                            NA 
                   rfi.scaled                    nfi.scaled 
                        0.965                         0.998 
                   ifi.scaled                    rni.scaled 
                        1.004                         1.004 
                   rni.robust                         rmsea 
                           NA                         0.000 
               rmsea.ci.lower                rmsea.ci.upper 
                        0.000                         0.190 
                 rmsea.pvalue                  rmsea.scaled 
                        0.596                         0.000 
        rmsea.ci.lower.scaled         rmsea.ci.upper.scaled 
                        0.000                         0.190 
          rmsea.pvalue.scaled                  rmsea.robust 
                        0.596                            NA 
        rmsea.ci.lower.robust         rmsea.ci.upper.robust 
                        0.000                            NA 
          rmsea.pvalue.robust                           rmr 
                           NA                         0.276 
                   rmr_nomean                          srmr 
                        0.100                         0.031 
                 srmr_bentler           srmr_bentler_nomean 
                        0.263                         0.031 
                         crmr                   crmr_nomean 
                        0.297                         0.028 
                   srmr_mplus             srmr_mplus_nomean 
                           NA                            NA 
                        cn_05                         cn_01 
                     1361.841                      2351.419 
                          gfi                          agfi 
                        1.000                         0.992 
                         pgfi                           mfi 
                        0.032                         1.002
```

The full output of this model, with all the fit indices, and including only assumed causal arrows, is:

```
lavaan 0.6-9 ended normally after 102 iterations

  Estimator                                       DWLS
  Optimization method                           NLMINB
  Number of model parameters                        30
                                                      
  Number of observations                           142
                                                      
Model Test User Model:
                                              Standard      Robust
  Test Statistic                                 0.398       0.398
  Degrees of freedom                                 1           1
  P-value (Chi-square)                           0.528       0.528
  Scaling correction factor                                  1.000
  Shift parameter                                            0.000
       simple second-order correction                             

Parameter Estimates:

  Standard errors                           Robust.sem
  Information                                 Expected
  Information saturated (h1) model        Unstructured

Regressions:
                   Estimate  Std.Err  z-value  P(>|z|)
  blue ~                                              
    uvb     (b_bu)   -0.756    0.342   -2.209    0.027
    d2l     (b_bd)   -0.132    0.051   -2.569    0.010
    subs    (b_bs)    0.226    0.135    1.669    0.095
    clim    (b_bc)   -0.007    0.212   -0.035    0.972
    lat     (b_bl)   -1.313    2.032   -0.646    0.518
    psiz    (b_bp)    0.090    0.025    3.564    0.000
  uvb ~                                               
    lat     (b_ul)   -5.671    0.151  -37.552    0.000
  clim ~                                              
    lat     (b_cl)    5.100    0.298   17.113    0.000
  d2l ~                                               
    lat     (b_dl)   -4.088    0.781   -5.233    0.000
  subs ~                                              
    lat     (b_sl)   -1.129    0.695   -1.625    0.104
  psiz ~                                              
    lat     (b_pl)  -10.916    3.255   -3.354    0.001
    clim    (b_pc)    2.435    0.540    4.512    0.000

Covariances:
                   Estimate  Std.Err  z-value  P(>|z|)
 .uvb ~~                                              
   .clim             -0.033    0.014   -2.447    0.014
   .d2l               0.070    0.038    1.840    0.066
 .clim ~~                                             
   .subs              0.008    0.067    0.127    0.899
 .subs ~~                                             
   .psiz              2.144    0.370    5.800    0.000
 .uvb ~~                                              
   .subs             -0.081    0.034   -2.420    0.016
 .d2l ~~                                              
   .subs             -0.386    0.190   -2.036    0.042
 .uvb ~~                                              
   .psiz             -0.185    0.080   -2.307    0.021
 .d2l ~~                                              
   .psiz             -1.703    0.479   -3.553    0.000

Intercepts:
                   Estimate  Std.Err  z-value  P(>|z|)
   .blue              0.000                           
   .uvb               0.927    0.047   19.793    0.000
   .clim             -0.834    0.056  -14.841    0.000
   .d2l               4.431    0.177   24.966    0.000
   .subs              0.000                           
   .psiz             15.564    0.682   22.807    0.000

Thresholds:
                   Estimate  Std.Err  z-value  P(>|z|)
    blue|t1           0.247    0.575    0.429    0.668
    subs|t1          -1.497    0.194   -7.739    0.000

Variances:
                   Estimate  Std.Err  z-value  P(>|z|)
   .blue              0.547                           
   .uvb               0.083    0.013    6.239    0.000
   .clim              0.257    0.029    8.756    0.000
   .d2l               2.542    0.333    7.625    0.000
   .subs              1.000                           
   .psiz             15.024    1.578    9.520    0.000

Scales y*:
                   Estimate  Std.Err  z-value  P(>|z|)
    blue              1.000                           
    subs              1.000
```

```
                         npar                          fmin 
                       30.000                         0.001 
                        chisq                            df 
                        0.398                         1.000 
                       pvalue                  chisq.scaled 
                        0.528                         0.398 
                    df.scaled                 pvalue.scaled 
                        1.000                         0.528 
         chisq.scaling.factor                baseline.chisq 
                        1.000                       215.483 
                  baseline.df               baseline.pvalue 
                       15.000                         0.000 
        baseline.chisq.scaled            baseline.df.scaled 
                      169.499                        15.000 
       baseline.pvalue.scaled baseline.chisq.scaling.factor 
                        0.000                         1.298 
                          cfi                           tli 
                        1.000                         1.045 
                         nnfi                           rfi 
                        1.045                         0.972 
                          nfi                          pnfi 
                        0.998                         0.067 
                          ifi                           rni 
                        1.003                         1.003 
                   cfi.scaled                    tli.scaled 
                        1.000                         1.058 
                   cfi.robust                    tli.robust 
                           NA                            NA 
                  nnfi.scaled                   nnfi.robust 
                        1.058                            NA 
                   rfi.scaled                    nfi.scaled 
                        0.965                         0.998 
                   ifi.scaled                    rni.scaled 
                        1.004                         1.004 
                   rni.robust                         rmsea 
                           NA                         0.000 
               rmsea.ci.lower                rmsea.ci.upper 
                        0.000                         0.190 
                 rmsea.pvalue                  rmsea.scaled 
                        0.596                         0.000 
        rmsea.ci.lower.scaled         rmsea.ci.upper.scaled 
                        0.000                         0.190 
          rmsea.pvalue.scaled                  rmsea.robust 
                        0.596                            NA 
        rmsea.ci.lower.robust         rmsea.ci.upper.robust 
                        0.000                            NA 
          rmsea.pvalue.robust                           rmr 
                           NA                         0.017 
                   rmr_nomean                          srmr 
                        0.014                         0.015 
                 srmr_bentler           srmr_bentler_nomean 
                        0.018                         0.015 
                         crmr                   crmr_nomean 
                        0.018                         0.014 
                   srmr_mplus             srmr_mplus_nomean 
                           NA                            NA 
                        cn_05                         cn_01 
                     1361.841                      2351.419 
                          gfi                          agfi 
                        1.000                         0.997 
                         pgfi                           mfi 
                        0.032                         1.002
```

## 8.3 Appendix III. Path analysis for “daltonism”

The full output of this model with only the 4 essential variables, with all the fit indices, is:

```
lavaan 0.6-9 ended normally after 34 iterations

  Estimator                                       DWLS
  Optimization method                           NLMINB
  Number of model parameters                        10
                                                      
  Number of observations                           142
                                                      
Model Test User Model:
                                              Standard      Robust
  Test Statistic                                 0.061       0.094
  Degrees of freedom                                 1           1
  P-value (Chi-square)                           0.804       0.759
  Scaling correction factor                                  0.649
  Shift parameter                                            0.000
       simple second-order correction                             

Parameter Estimates:

  Standard errors                           Robust.sem
  Information                                 Expected
  Information saturated (h1) model        Unstructured

Regressions:
                   Estimate  Std.Err  z-value  P(>|z|)
  blue ~                                              
    uvb     (b_bu)   -1.360    0.306   -4.450    0.000
    lat     (b_bl)   -0.044    0.018   -2.372    0.018
  uvb ~                                               
    lat     (b_ul)   -0.057    0.002  -37.533    0.000
  dalt ~                                              
    uvb     (b_du)   -0.872    0.185   -4.704    0.000
    blue    (b_db)    0.719    0.212    3.387    0.001

Intercepts:
                   Estimate  Std.Err  z-value  P(>|z|)
   .blue              0.000                           
   .uvb               0.927    0.047   19.793    0.000
   .dalt              4.383    0.304   14.432    0.000

Thresholds:
                   Estimate  Std.Err  z-value  P(>|z|)
    blue|t1          -0.952    0.326   -2.924    0.003

Variances:
                   Estimate  Std.Err  z-value  P(>|z|)
   .blue              0.848                           
   .uvb               0.082    0.013    6.156    0.000
   .dalt              4.005    0.484    8.269    0.000

Scales y*:
                   Estimate  Std.Err  z-value  P(>|z|)
    blue              1.000
```

```
                         npar                          fmin 
                       10.000                         0.000 
                        chisq                            df 
                        0.061                         1.000 
                       pvalue                  chisq.scaled 
                        0.804                         0.094 
                    df.scaled                 pvalue.scaled 
                        1.000                         0.759 
         chisq.scaling.factor                baseline.chisq 
                        0.649                        43.669 
                  baseline.df               baseline.pvalue 
                        3.000                         0.000 
        baseline.chisq.scaled            baseline.df.scaled 
                       41.011                         3.000 
       baseline.pvalue.scaled baseline.chisq.scaling.factor 
                        0.000                         1.070 
                          cfi                           tli 
                        1.000                         1.069 
                         nnfi                           rfi 
                        1.069                         0.996 
                          nfi                          pnfi 
                        0.999                         0.333 
                          ifi                           rni 
                        1.022                         1.023 
                   cfi.scaled                    tli.scaled 
                        1.000                         1.071 
                   cfi.robust                    tli.robust 
                           NA                            NA 
                  nnfi.scaled                   nnfi.robust 
                        1.071                            NA 
                   rfi.scaled                    nfi.scaled 
                        0.993                         0.998 
                   ifi.scaled                    rni.scaled 
                        1.023                         1.024 
                   rni.robust                         rmsea 
                           NA                         0.000 
               rmsea.ci.lower                rmsea.ci.upper 
                        0.000                         0.141 
                 rmsea.pvalue                  rmsea.scaled 
                        0.835                         0.000 
        rmsea.ci.lower.scaled         rmsea.ci.upper.scaled 
                        0.000                         0.152 
          rmsea.pvalue.scaled                  rmsea.robust 
                        0.796                            NA 
        rmsea.ci.lower.robust         rmsea.ci.upper.robust 
                        0.000                            NA 
          rmsea.pvalue.robust                           rmr 
                           NA                         0.423 
                   rmr_nomean                          srmr 
                        0.058                         0.025 
                 srmr_bentler           srmr_bentler_nomean 
                        0.421                         0.025 
                         crmr                   crmr_nomean 
                        0.515                         0.032 
                   srmr_mplus             srmr_mplus_nomean 
                           NA                            NA 
                        cn_05                         cn_01 
                     8837.596                     15263.405 
                          gfi                          agfi 
                        1.000                         0.998 
                         pgfi                           mfi 
                        0.091                         1.003
```

The full output of this model with only the 4 essential variables and *subsistence*, with all the fit indices, is:

```
lavaan 0.6-9 ended normally after 46 iterations

  Estimator                                       DWLS
  Optimization method                           NLMINB
  Number of model parameters                        15
                                                      
  Number of observations                           142
                                                      
Model Test User Model:
                                              Standard      Robust
  Test Statistic                                 1.167       2.398
  Degrees of freedom                                 1           1
  P-value (Chi-square)                           0.280       0.122
  Scaling correction factor                                  0.487
  Shift parameter                                           -0.000
       simple second-order correction                             

Parameter Estimates:

  Standard errors                           Robust.sem
  Information                                 Expected
  Information saturated (h1) model        Unstructured

Regressions:
                   Estimate  Std.Err  z-value  P(>|z|)
  blue ~                                              
    uvb     (b_bu)   -1.019    0.356   -2.859    0.004
    lat     (b_bl)   -0.019    0.021   -0.928    0.354
    subs    (b_bs)    0.473    0.119    3.972    0.000
  uvb ~                                               
    lat     (b_ul)   -0.057    0.002  -37.496    0.000
  dalt ~                                              
    uvb     (b_du)   -1.354    0.211   -6.401    0.000
    blue    (b_db)    0.168    0.233    0.722    0.470
    subs    (b_ds)    0.997    0.249    4.007    0.000
  subs ~                                              
    lat     (b_sl)   -0.010    0.007   -1.496    0.135

Covariances:
                   Estimate  Std.Err  z-value  P(>|z|)
 .uvb ~~                                              
   .subs             -0.066    0.034   -1.954    0.051

Intercepts:
                   Estimate  Std.Err  z-value  P(>|z|)
   .blue              0.000                           
   .uvb               0.927    0.047   19.793    0.000
   .dalt              4.082    0.231   17.677    0.000
   .subs              0.000                           

Thresholds:
                   Estimate  Std.Err  z-value  P(>|z|)
    blue|t1          -0.635    0.366   -1.737    0.082
    subs|t1          -1.497    0.194   -7.739    0.000

Variances:
                   Estimate  Std.Err  z-value  P(>|z|)
   .blue              0.854                           
   .uvb               0.079    0.013    6.071    0.000
   .dalt              3.214    0.517    6.220    0.000
   .subs              1.000                           

Scales y*:
                   Estimate  Std.Err  z-value  P(>|z|)
    blue              1.000                           
    subs              1.000
```

```
                         npar                          fmin 
                       15.000                         0.004 
                        chisq                            df 
                        1.167                         1.000 
                       pvalue                  chisq.scaled 
                        0.280                         2.398 
                    df.scaled                 pvalue.scaled 
                        1.000                         0.122 
         chisq.scaling.factor                baseline.chisq 
                        0.487                       111.099 
                  baseline.df               baseline.pvalue 
                        6.000                         0.000 
        baseline.chisq.scaled            baseline.df.scaled 
                       98.537                         6.000 
       baseline.pvalue.scaled baseline.chisq.scaling.factor 
                        0.000                         1.136 
                          cfi                           tli 
                        0.998                         0.990 
                         nnfi                           rfi 
                        0.990                         0.937 
                          nfi                          pnfi 
                        0.989                         0.165 
                          ifi                           rni 
                        0.998                         0.998 
                   cfi.scaled                    tli.scaled 
                        0.985                         0.909 
                   cfi.robust                    tli.robust 
                           NA                            NA 
                  nnfi.scaled                   nnfi.robust 
                        0.909                            NA 
                   rfi.scaled                    nfi.scaled 
                        0.854                         0.976 
                   ifi.scaled                    rni.scaled 
                        0.986                         0.985 
                   rni.robust                         rmsea 
                           NA                         0.034 
               rmsea.ci.lower                rmsea.ci.upper 
                        0.000                         0.229 
                 rmsea.pvalue                  rmsea.scaled 
                        0.360                         0.100 
        rmsea.ci.lower.scaled         rmsea.ci.upper.scaled 
                        0.000                         0.269 
          rmsea.pvalue.scaled                  rmsea.robust 
                        0.186                            NA 
        rmsea.ci.lower.robust         rmsea.ci.upper.robust 
                        0.000                            NA 
          rmsea.pvalue.robust                           rmr 
                           NA                         0.260 
                   rmr_nomean                          srmr 
                        0.075                         0.079 
                 srmr_bentler           srmr_bentler_nomean 
                        0.261                         0.079 
                         crmr                   crmr_nomean 
                        0.274                         0.054 
                   srmr_mplus             srmr_mplus_nomean 
                           NA                            NA 
                        cn_05                         cn_01 
                      465.077                       802.546 
                          gfi                          agfi 
                        0.999                         0.991 
                         pgfi                           mfi 
                        0.062                         0.999
```

# 9 Session information

**CPU:** AMD Ryzen 7 3700X 8-Core Processor (16 threads)

**RAM (memory):** 67.5 GB

**R version 4.0.5 (2021-03-31)**

**Platform:** x86\_64-pc-linux-gnu (64-bit)

**locale:** *LC\_CTYPE=en\_US.UTF-8*, *LC\_NUMERIC=C*, *LC\_TIME=en\_US.UTF-8*, *LC\_COLLATE=en\_US.UTF-8*, *LC\_MONETARY=en\_US.UTF-8*, *LC\_MESSAGES=en\_US.UTF-8*, *LC\_PAPER=en\_US.UTF-8*, *LC\_NAME=C*, *LC\_ADDRESS=C*, *LC\_TELEPHONE=C*, *LC\_MEASUREMENT=en\_US.UTF-8* and *LC\_IDENTIFICATION=C*

**attached base packages:** *grid*, *parallel*, *stats*, *graphics*, *grDevices*, *utils*, *datasets*, *methods* and *base*

**other attached packages:** *benchmarkme(v.1.0.7)*, *rminer(v.1.4.6)*, *rsample(v.0.1.0)*, *randomForest(v.4.6-14)*, *partykit(v.1.2-13)*, *mvtnorm(v.1.1-2)*, *libcoin(v.1.0-8)*, *caret(v.6.0-88)*, *lattice(v.0.20-44)*, *semPlot(v.1.1.2)*, *lavaanPlot(v.0.5.1)*, *lavaan(v.0.6-9)*, *performance(v.0.7.2)*, *sjstats(v.0.18.1)*, *bayestestR(v.0.10.0)*, *brms(v.2.15.0)*, *Rcpp(v.1.0.7)*, *lmerTest(v.3.1-3)*, *lme4(v.1.1-27.1)*, *Matrix(v.1.3-4)*, *maps(v.3.3.0)*, *geosphere(v.1.5-10)*, *Imap(v.1.32)*, *igraph(v.1.2.6)*, *deldir(v.0.2-10)*, *spdep(v.1.1-8)*, *sf(v.1.0-1)*, *spData(v.0.3.10)*, *ape(v.5.5)*, *scanstatistics(v.1.0.1)*, *spatstat(v.2.2-0)*, *spatstat.linnet(v.2.2-1)*, *spatstat.core(v.2.2-0)*, *rpart(v.4.1-15)*, *nlme(v.3.1-152)*, *spatstat.geom(v.2.2-0)*, *spatstat.data(v.2.1-0)*, *maptools(v.1.1-1)*, *sp(v.1.4-5)*, *DiagrammeR(v.1.0.6.1)*, *ggnewscale(v.0.4.5)*, *gridExtra(v.2.3)*, *ggrepel(v.0.9.1)*, *ggplot2(v.3.3.5)*, *reshape2(v.1.4.4)*, *dplyr(v.1.0.7)*, *plyr(v.1.8.6)*, *pbapply(v.1.4-3)*, *future(v.1.21.0)*, *pander(v.0.6.4)*, *knitr(v.1.33)* and *RhpcBLASctl(v.0.20-137)*

**loaded via a namespace (and not attached):** *Hmisc(v.4.5-0)*, *corpcor(v.1.6.9)*, *class(v.7.3-19)*, *ps(v.1.6.0)*, *glmnet(v.4.1-2)*, *foreach(v.1.5.1)*, *projpred(v.2.0.2)*, *crayon(v.1.4.1)*, *V8(v.3.4.2)*, *MASS(v.7.3-54)*, *backports(v.1.2.1)*, *colourpicker(v.1.1.0)*, *rlang(v.0.4.11)*, *nloptr(v.1.2.2.2)*, *callr(v.3.7.0)*, *strucchange(v.1.5-2)*, *xgboost(v.1.4.1.1)*, *sets(v.1.0-18)*, *reliaR(v.0.01)*, *glue(v.1.4.2)*, *loo(v.2.4.1)*, *rstan(v.2.21.2)*, *processx(v.3.5.2)*, *spatstat.sparse(v.2.0-0)*, *classInt(v.0.4-3)*, *tidyselect(v.1.1.1)*, *XML(v.3.99-0.6)*, *tidyr(v.1.1.3)*, *zoo(v.1.8-9)*, *sjmisc(v.2.8.7)*, *xtable(v.1.8-4)*, *regsem(v.1.8.0)*, *magrittr(v.2.0.1)*, *evaluate(v.0.14)*, *cli(v.3.0.0)*, *rstudioapi(v.0.13)*, *miniUI(v.0.1.1.1)*, *furrr(v.0.2.3)*, *bslib(v.0.2.5.1)*, *pls(v.2.7-3)*, *sjlabelled(v.1.1.8)*, *shinystan(v.2.5.0)*, *shiny(v.1.6.0)*, *xfun(v.0.24)*, *parameters(v.0.14.0)*, *inline(v.0.3.19)*, *pkgbuild(v.1.2.0)*, *cluster(v.2.1.2)*, *bridgesampling(v.1.1-2)*, *tibble(v.3.1.2)*, *expm(v.0.999-6)*, *Brobdingnag(v.1.2-6)*, *threejs(v.0.3.3)*, *listenv(v.0.8.0)*, *png(v.0.1-7)*, *ipred(v.0.9-11)*, *withr(v.2.4.2)*, *e1071(v.1.7-7)*, *pROC(v.1.17.0.1)*, *coda(v.0.19-4)*, *pillar(v.1.6.1)*, *RcppParallel(v.5.1.4)*, *multcomp(v.1.4-17)*, *kernlab(v.0.9-29)*, *raster(v.3.4-13)*, *gmodels(v.2.18.1)*, *xts(v.0.12.1)*, *vctrs(v.0.3.8)*, *pbivnorm(v.0.6.0)*, *ellipsis(v.0.3.2)*, *generics(v.0.1.0)*, *dygraphs(v.1.1.1.6)*, *lava(v.1.6.9)*, *rgdal(v.1.5-23)*, *mda(v.0.5-2)*, *tools(v.4.0.5)*, *foreign(v.0.8-81)*, *munsell(v.0.5.0)*, *gamm4(v.0.2-6)*, *Cubist(v.0.3.0)*, *emmeans(v.1.6.2-1)*, *proxy(v.0.4-26)*, *fastmap(v.1.1.0)*, *compiler(v.4.0.5)*, *abind(v.1.4-5)*, *httpuv(v.1.6.1)*, *prodlim(v.2019.11.13)*, *kknn(v.1.3.1)*, *OpenMx(v.2.19.6)*, *visNetwork(v.2.0.9)*, *utf8(v.1.2.1)*, *later(v.1.2.0)*, *recipes(v.0.1.16)*, *jsonlite(v.1.7.2)*, *arm(v.1.11-2)*, *scales(v.1.1.1)*, *carData(v.3.0-4)*, *estimability(v.1.3)*, *LearnBayes(v.2.15.1)*, *promises(v.1.2.0.1)*, *doParallel(v.1.0.16)*, *latticeExtra(v.0.6-29)*, *goftest(v.1.2-2)*, *spatstat.utils(v.2.2-0)*, *effectsize(v.0.4.5)*, *checkmate(v.2.0.0)*, *rmarkdown(v.2.9)*, *openxlsx(v.4.2.4)*, *sandwich(v.3.0-1)*, *survival(v.3.2-11)*, *numDeriv(v.2016.8-1.1)*, *rsconnect(v.0.8.18)*, *yaml(v.2.2.1)*, *plotrix(v.3.8-1)*, *bayesplot(v.1.8.1)*, *htmltools(v.0.5.1.1)*, *rstantools(v.2.1.1)*, *modeltools(v.0.2-23)*, *viridisLite(v.0.4.0)*, *digest(v.0.6.27)*, *assertthat(v.0.2.1)*, *mime(v.0.11)*, *emdbook(v.1.3.12)*, *units(v.0.7-2)*, *rockchalk(v.1.8.144)*, *data.table(v.1.14.0)*, *ismev(v.1.42)*, *labeling(v.0.4.2)*, *shinythemes(v.1.2.0)*, *splines(v.4.0.5)*, *Formula(v.1.2-4)*, *broom(v.0.7.8)*, *modelr(v.0.1.8)*, *colorspace(v.2.0-2)*, *adabag(v.4.2)*, *base64enc(v.0.1-3)*, *mnormt(v.2.0.2)*, *shape(v.1.4.6)*, *tmvnsim(v.1.0-2)*, *inum(v.1.0-4)*, *nnet(v.7.3-16)*, *sass(v.0.4.0)*, *coin(v.1.4-1)*, *matrixcalc(v.1.0-4)*, *fansi(v.0.5.0)*, *truncnorm(v.1.0-8)*, *parallelly(v.1.26.1)*, *ModelMetrics(v.1.2.2.2)*, *R6(v.2.5.0)*, *ggridges(v.0.5.3)*, *lifecycle(v.1.0.0)*, *StanHeaders(v.2.21.0-7)*, *zip(v.2.2.0)*, *curl(v.4.3.2)*, *minqa(v.1.2.4)*, *gdata(v.2.18.0)*, *mi(v.1.0)*, *jquerylib(v.0.1.4)*, *benchmarkmeData(v.1.0.4)*, *party(v.1.3-7)*, *qgraph(v.1.6.9)*, *glasso(v.1.11)*, *TH.data(v.1.0-10)*, *RColorBrewer(v.1.1-2)*, *iterators(v.1.0.13)*, *stringr(v.1.4.0)*, *gower(v.0.2.2)*, *htmlwidgets(v.1.5.3)*, *polyclip(v.1.10-0)*, *markdown(v.1.1)*, *purrr(v.0.3.4)*, *crosstalk(v.1.1.1)*, *mgcv(v.1.8-36)*, *globals(v.0.14.0)*, *insight(v.0.14.2)*, *htmlTable(v.2.2.1)*, *bdsmatrix(v.1.3-4)*, *codetools(v.0.2-18)*, *matrixStats(v.0.59.0)*, *lubridate(v.1.7.10)*, *gtools(v.3.9.2)*, *prettyunits(v.1.1.1)*, *psych(v.2.1.6)*, *gtable(v.0.3.0)*, *DBI(v.1.1.1)*, *stats4(v.4.0.5)*, *httr(v.1.4.2)*, *highr(v.0.9)*, *tensor(v.1.5)*, *KernSmooth(v.2.23-20)*, *stringi(v.1.6.2)*, *farver(v.2.1.0)*, *fdrtool(v.1.2.16)*, *timeDate(v.3043.102)*, *DT(v.0.18)*, *lisrelToR(v.0.1.4)*, *bbmle(v.1.0.23.1)*, *boot(v.1.3-28)*, *shinyjs(v.2.0.0)*, *sem(v.3.1-11)*, *kutils(v.1.70)*, *jpeg(v.0.1-8.1)*, *pkgconfig(v.2.0.3)* and *Rsolnp(v.1.16)*

# References

Adam, A. (1969). A further query on color blindness and natural selection. *Social Biology*, *16*(3), 197–202. https://doi.org/10.1080/19485565.1969.9987819

Baddeley, A., Rubak, E., & Turner, R. (2015). *Spatial point patterns: Methodology and applications with R*. Retrieved from http://www.crcpress.com/Spatial-Point-Patterns-Methodology-and-Applications-with-R/Baddeley-Rubak-Turner/9781482210200/

Bentz, C., Dediu, D., Verkerk, A., & Jäger, G. (2018). The evolution of language families is shaped by the environment beyond neutral drift. *Nature Human Behaviour*, *2*(11), 816. https://doi.org/10.1038/s41562-018-0457-6

Berlin, B., & Kay, P. (1991). *Basic Color Terms: Their Universality and Evolution*. University of California Press.

Bickel, B., Nichols, J., Zakharko, T., Witzlack-Makarevich, A., Hildebrandt, K., Rießler, M., … Lowe, J. B. (2017). *The AUTOTYP typological databases (Version 0.1.0)*. Retrieved from https://github.com/autotyp/autotyp-data/tree/0.1.0

Blasi, D. E., Moran, S., Moisik, S. R., Widmer, P., Dediu, D., & Bickel, B. (2019). Human sound systems are shaped by post-Neolithic changes in bite configuration. *Science*, *363*(6432), eaav3218. https://doi.org/10.1126/science.aav3218

Brown, A. M., & Lindsey, D. T. (2004). Color and language: Worldwide distribution of Daltonism and distinct words for "blue". *Visual Neuroscience*, *21*(3), 409–412. Retrieved from http://www.ncbi.nlm.nih.gov/pubmed/15518222

Cysouw, M., Dediu, D., & Moran, S. (2012). Comment on “Phonemic Diversity Supports a Serial Founder Effect Model of Language Expansion from Africa.” *Science*, *335*(6069), 657. https://doi.org/10.1126/science.1208841

De Soete, G. (1984). Ultrametric tree representations of incomplete dissimilarity data. *Journal of Classification*, *1*(1), 235–242. https://doi.org/10.1007/BF01890124

Dediu, D., Janssen, R., & Moisik, S. R. (2017). Language is not isolated from its wider environment: Vocal tract influences on the evolution of speech and language. *Language and Communication*, *54*, 9–20. https://doi.org/doi:10.1016/j.langcom.2016.10.002

Dediu, D., & Ladd, D. R. (2007). Linguistic tone is related to the population frequency of the adaptive haplogroups of two brain size genes, ASPM and Microcephalin. *Proc Natl Acad Sci U S A*, *104*(26), 10944–10949. https://doi.org/10.1073/pnas.0610848104

Dediu, D., & Moisik, S. R. (2019). Pushes and pulls from below: Anatomical variation, articulation and sound change. *Glossa: A Journal of General Linguistics*, *4*(1), 7. https://doi.org/10.5334/gjgl.646

Excoffier, L., & Lischer, H. E. L. (2010). Arlequin suite ver 3.5: A new series of programs to perform population genetics analyses under Linux and Windows. *Molecular Ecology Resources*, *10*(3), 564–567. https://doi.org/10.1111/j.1755-0998.2010.02847.x

Garth, T. R. (1933). THE INCIDENCE OF COLOR BLINDNESS AMONG RACES. *Science*, *77*(1996), 333–334. https://doi.org/10.1126/science.77.1996.333

Grace, J. B., & Bollen, K. A. (2005). Interpreting the Results from Multiple Regression and Structural Equation Models. *The Bulletin of the Ecological Society of America*, *86*(4), 283–295. https://doi.org/10.1890/0012-9623(2005)86[283:ITRFMR]2.0.CO;2

Hammarström, H., Bank, S., Forkel, R., & Haspelmath, M. (2018). *Glottolog 3.2*. Retrieved from http://glottolog.org

Hardy, L. H., Rand, G., & Rittler, M. C. (1954). HRR polychromatic plates. *JOSA*, *44*(7), 509–521.

Holsinger, K. E., & Weir, B. S. (2009). Genetics in geographically structured populations: Defining, estimating and interpreting F(ST). *Nature Reviews. Genetics*, *10*(9), 639–650. https://doi.org/10.1038/nrg2611

Ishihara, S. (1917). *Tests for color-blindness*. Tokyo: Hongo Harukicho.

Josserand, M. (2020). *Speaking about colors: A cross-linguistic statistical investigation of the effects of the physical environment on the way languages conceptualize the color space* (Master’s thesis). Ecole Normale Supérieure, Lyon, France.

Junqueira, P. C., Kalmus, H., & Wishart, P. (1957). P.T.C. THRESHOLDS, COLOUR VISION AND BLOOD FACTORS OF BRAZILIAN INDIANS.: CARAJAS. *Annals of Human Genetics*, *22*(1), 22–25. https://doi.org/10.1111/j.1469-1809.1957.tb01295.x

Kalmus, H. (1957). DEFECTIVE COLOUR VISION, P.T.C. TASTING AND DREPANOCYTOSIS IN SAMPLES FROM FIFTEEN BRAZILIAN POPULATIONS. *Annals of Human Genetics*, *21*(4), 313–317. https://doi.org/10.1111/j.1469-1809.1972.tb00202.x

Kalmus, H., Amir, A., Levine, O., Barak, E., & Goldschmidt, E. (1961). The frequency of inherited defects of colour vision in some Israeli populations. *Annals of Human Genetics*, *25*(1), 51–55. https://doi.org/10.1111/j.1469-1809.1961.tb01496.x

Kalmus, H., Degaray, A. L., Rodarte, U., & Cobo, L. (1964). THE FREQUENCY OF PTC TASTING HARD EAR WAX, COLOUR BLINDNESS AND OTHER GENETICAL CHARACTERS IN URBAN AND RURAL MEXICAN POPULATIONS. *Human Biology*, *36*, 134–145.

Kirby, K. R., Gray, R. D., Greenhill, S. J., Jordan, F. M., Gomes-Ng, S., Bibiko, H.-J., … Gavin, M. C. (2016). D-PLACE: A Global Database of Cultural, Linguistic and Environmental Diversity. *PLOS ONE*, *11*(7), e0158391. https://doi.org/10.1371/journal.pone.0158391

Knoll, H. A. (1968). *Patent No. US3382025A*. Retrieved from https://patents.google.com/patent/US3382025A/en

Lapointe, F. J., & Kirsch, J. a. W. (1995). Estimating Phylogenies from Lacunose Distance Matrices, with Special Reference to DNA Hybridization Data. *Molecular Biology and Evolution*, *12*(2), 266–266. https://doi.org/10.1093/oxfordjournals.molbev.a040209

Lewis, M. P. (Ed.). (2014). *Ethnologue: Languages of the World* (17th ed.). Retrieved from http://www.ethnologue.com/

Lindsey, D. T., & Brown, A. M. (2002). Color Naming and the Phototoxic Effects of Sunlight on the Eye. *Psychological Science*, *13*(6), 506–512. https://doi.org/10.1111/1467-9280.00489

Meeussen, E. (2015). *Colour blindness and its contribution to colour vocabulary* (Master’s thesis). Radboud Universiteit Nijmegen, Nijmegen, The Netherlands.

Moisik, S. R., & Dediu, D. (2017). Anatomical biasing and clicks: Evidence from biomechanical modeling. *Journal of Language Evolution*, *2*(1), 37–51. https://doi.org/doi:10.1093/jole/lzx004

Moran, P. A. P. (1950). Notes on Continuous Stochastic Phenomena. *Biometrika*, *37*(1/2), 17–23. https://doi.org/10.2307/2332142

Oldoni, F., Kidd, K. K., & Podini, D. (2019). Microhaplotypes in forensic genetics. *Forensic Science International. Genetics*, *38*, 54–69. https://doi.org/10.1016/j.fsigen.2018.09.009

Pickford, R. W. (1963). Natural selection and colour blindness. *The Eugenics Review*, *55*(2), 97–101.

Post, R. H. (1962). Population differences in red and green color vision deficiency: A review, and a query on selection relaxation. *Eugenics Quarterly*, *9*, 131–146. https://doi.org/10.1080/19485565.1962.9987517

Post, Richard H. (1963). ‘Colorblindness” and relaxed selection. *Eugenics Quarterly*, *10*(2), 84–85. https://doi.org/10.1080/19485565.1963.9987549

R Core Team. (2021). *R: A language and environment for statistical computing*. Retrieved from https://www.R-project.org/

Rajeevan, H. (2003). ALFRED: The ALelle FREquency Database. Update. *Nucleic Acids Research*, *31*(1), 270–271. https://doi.org/10.1093/nar/gkg043

Salzano, F. M. (1961). Rare genetic conditions among the Caingang Indians. *Annals of Human Genetics*, *25*, 123–130. https://doi.org/10.1111/j.1469-1809.1961.tb01509.x

Salzano, F. M. (1964). COLOR BLINDNESS AMONG INDIANS FROM SANTA CATARINA, BRAZIL. *Acta Genetica Et Statistica Medica*, *14*, 212–219. https://doi.org/10.1159/000151848

Spielman, S. E. (2017). Point Pattern Analysis. In *International Encyclopedia of Geography* (pp. 1–9). https://doi.org/10.1002/9781118786352.wbieg0849

Thomson, W. (1880). An instrument for the detection of color blindness. *Transactions of the American Ophthalmological Society*, *3*, 142.

Turchin, P., Brennan, R., Currie, T., Feeney, K., Francois, P., Hoyer, D., … Whitehouse, H. (2015). Seshat: The Global History Databank. *Cliodynamics*, *6*(1). https://doi.org/10.21237/C7clio6127917

Wichmann, S., Müller, A., & Velupillai, V. (2010). Homelands of the world’s language families: A quantitative approach. *Diachronica*, *27*(2), 247–276. https://doi.org/10.1075/dia.27.2.05wic

---

1. This is an example note. Click the symbol at the end of the note to go back to where the note is called in the text.↩︎
2. Please note that some of the models used here are computationally expensive, and even compiling this `Rmarkdown` script might require a relatively powerful machine. To help with this, and to ensure full replicability of our results, we have cached some of these expensive sections in the `cached_results` folder as `XZ`-compressed `RData` files. However, it might happen that versions of some of the packages different from those that we used here might not be fully compatible with the saved `RData` files, resulting in errors compiling this `Rmarkdown` script or errors displaying/plotting the results. In this case, we recommend using the exact same versions of `R` and of the packages that we used (listed in the *Session information*), or, if not possible, the deletion of the offending `RData` files and the full recompilation of the `Rmarkdown` script (which is smart enough to re-generate only those missing cached results).↩︎
3. Unfortunately, the primary data from OpenStreetMap, the “Reduced waterbodies as raster masks” seems to no longer be available for download as of July 2020, so we used here the original data downloaded in March 2018 by Dan Dediu and used in the Bentz, Dediu, Verkerk, & Jäger (2018) paper.↩︎
4. Unfortunately, the primary data from TOMS seems to no longer be available for download as of July 2020, so we used here the original data downloaded in 2012 by Dan Dediu and used in the Meeussen (2015) MSc thesis.↩︎
5. For details about interpreting the results of a path analysis or SEM model, please see here, here, here or here, but the main ideas are that the *χ*2 test should **not** be significant, that the various *fit indices* (such as the Comparative Fit Index (CFI), the Tucker-Lewis Index (TLI), and the Non-normed Fit Index (NNFI)) should be as close to 1.0 as possible (ok if ≥ 0.90), and that the Root Mean Square Error of Approximation (RMSEA) should be as small as possible (≤ 0.05) with a narrow 90% confidence interval (ideally with the lower limit very close to 0.0 and the upper limit ≤ 0.08).↩︎
6. A *Single Nucleotide Polymorphism* (*SNP*) is a common and simple type of genetic variation representing the substitution of a single nucleotide at a specific position in the genome.↩︎
7. A *haplotype* has several related meaning, in principle refering to a set of genetic variants that tend to be inherited together; a *microhaplotype* (microhap or *MH*) is a set of small set of closely linked SNPs useful in forensic applications (Oldoni, Kidd, & Podini, 2019).↩︎
